# Tracing local sources and long-range transport of PM_10_ in central Taiwan by using chemical characteristics and Pb isotope ratios

**DOI:** 10.1038/s41598-021-87051-y

**Published:** 2021-04-07

**Authors:** Po-Chao Wu, Kuo-Fang Huang

**Affiliations:** 1 Earth System Science Program, Taiwan International Graduate Program (TIGP), Academia Sinica, Taipei, Taiwan; 2 Institute of Earth Sciences, Academia Sinica, Taipei, Taiwan; 3 College of Earth Sciences, National Central University, Taoyuan, Taiwan

**Keywords:** Atmospheric chemistry, Environmental monitoring, Geochemistry

## Abstract

Central Taiwan is among the most heavily polluted regions in Taiwan because of a complex mixing of local emissions from intense anthropogenic activities with natural dust. Long-range transport (LRT) of pollutants from outside Taiwan also contributes critically to the deterioration of air quality, especially during the northeast monsoon season. To identify the sources of particulate matter < 10 μm (PM_10_) in central Taiwan, this study performed several sampling campaigns, including three local events, one LRT event, and one dust storm event, during the northeast monsoon season of 2018/2019. The PM_10_ samples were analyzed for water-soluble ion and trace metal concentrations as well as Pb isotope ratios. Local sediments were also collected and analyzed to constrain chemical/isotopic signatures of natural sources. The Pb isotope data were interpreted together with the enrichment factors and elemental ratios of trace metals in PM_10_, and reanalysis data sets were used to delineate the sources of PM_10_ in central Taiwan. Our results suggested that Pb in PM_10_ was predominantly contributed by oil combustion and oil refineries during the local events (48–88%), whereas the lowest contributions were from coal combustion (< 21%). During periods of high wind speed, the contribution from natural sources increased significantly from 13 to 31%. Despite Pb represented only a small portion of PM_10_, a strong correlation (r = 0.89, *p*  < 0.001, multiple regression analysis) between PM_10_ mass and the concentrations of Pb, V, and Al was observed in the study area, suggesting that the sources of PM_10_ in central Taiwan can be possibly tracked by using chemical characteristics and Pb isotopes in PM_10_. Moreover, the Pb isotopic signals of PM_10_ collected during the LRT event confirmed the impact of LRT from Mainland China, and the chemical characteristics of the PM_10_ significantly differed from those of the PM_10_ collected during local events. This study demonstrates the robustness of using a combination of Pb isotopic compositions and chemical characteristics in PM_10_ for source tracing in complex and heavily polluted areas.

## Introduction

Atmospheric particulate matter (PM) is derived from both natural and anthropogenic sources. It affects the atmospheric condition by reducing visibility and deteriorating air quality, and it even changes the Earth’s surface albedo, which influences regional climate changes^1,2^. In addition, high concentrations of PM, the main carrier of heavy metals, can cause lung function decline and increase the risk of respiratory and cardiovascular diseases^3–5^. For example, chronic exposure to Pb can be harmful to the neural system, leading to lower memory capabilities and even inducing cancer^6,7^. Thus, studying the sources of PM is vital for strategically mitigating such pollution.

In the recent three decades, emissions of anthropogenic PM and heavy metals into the atmosphere have been increasing in East Asia (e.g., PM_10_ emission increased from 20 Tg in 1990 to 27 Tg in 2010) as a result of growing economies and the rapid development of cities^8^. According to available global estimates, China is a major contributor of global PM and heavy metal emissions^8–10^. Despite a recent declining trend, atmospheric emissions are still the highest among Asian countries^9,11,12^. The influences of industrial aerosols from China through long-range transport (LRT) have been recorded in many areas, such as South Korea^13^, Japan^14,15^, the North Pacific^16^, and even the United States^17,18^ and Canada^19^. Taiwan, as a neighboring country approximately 100 miles away, has continued to receive airborne PM from China (including particles derived from anthropogenic activities and dust storms), particularly during the northeastern monsoon season^20–23^.

In addition to LRT, the coastal area of central Taiwan is also impacted by intense local anthropogenic activities, such as emissions from industrial parks, coal-fired power plants, petrochemical complexes, and vehicle exhaust. Studies have conducted detailed investigations into the chemical characteristics of PM < 2.5 μm (PM_2.5_) and PM < 10 μm (PM_10_) in this region^24–26^. Hsu et al*.*^25^ found that average PM_10_ concentrations were high (76.4 ± 22 μg m^−3^) during winter, with an annual PM_10_ concentration of 52.4 ± 27.2 μg m^−3^. Moreover, extremely high PM_10_ episodes (> 125 μg m^−3^) have also occurred occasionally during the northeast monsoon season^24,27^. The PM_10_ in central Taiwan was estimated using a receptor model derived from soil dust, crustal materials, coal combustion, oil combustion, and traffic emissions^25^. Although several studies have investigated the characteristics of PM and the contributions from local sources in central Taiwan, remarkably few have attempted to distinguish the chemical characteristics and Pb isotope ratios of PM_10_ from local sources and LRT.

The Pb isotope ratio is an essential tool for tracking pollution sources in the atmosphere. Pb exists in both natural and anthropogenic sources and has four naturally occurring isotopes: ^204^Pb is a non-radiogenic nucleus, whereas ^206^Pb, ^207^Pb, and ^208^Pb are radiogenic end products from the decay series of ^238^U, ^235^U, and ^232^Th, respectively. Pb is produced and released into the environment through human activities that use various ore minerals (e.g., Pb ore) with distinct Pb isotope ratios formed under varying geological conditions. These isotope ratios do not fractionate during industrial processes, making them a promising tool for identifying pollution sources^28,29^. This technique has been deployed to study PM sources in the atmosphere^14,18,30–33^ as well as the sources of particles in ice cores^29,34,35^.

**Figure 1:**
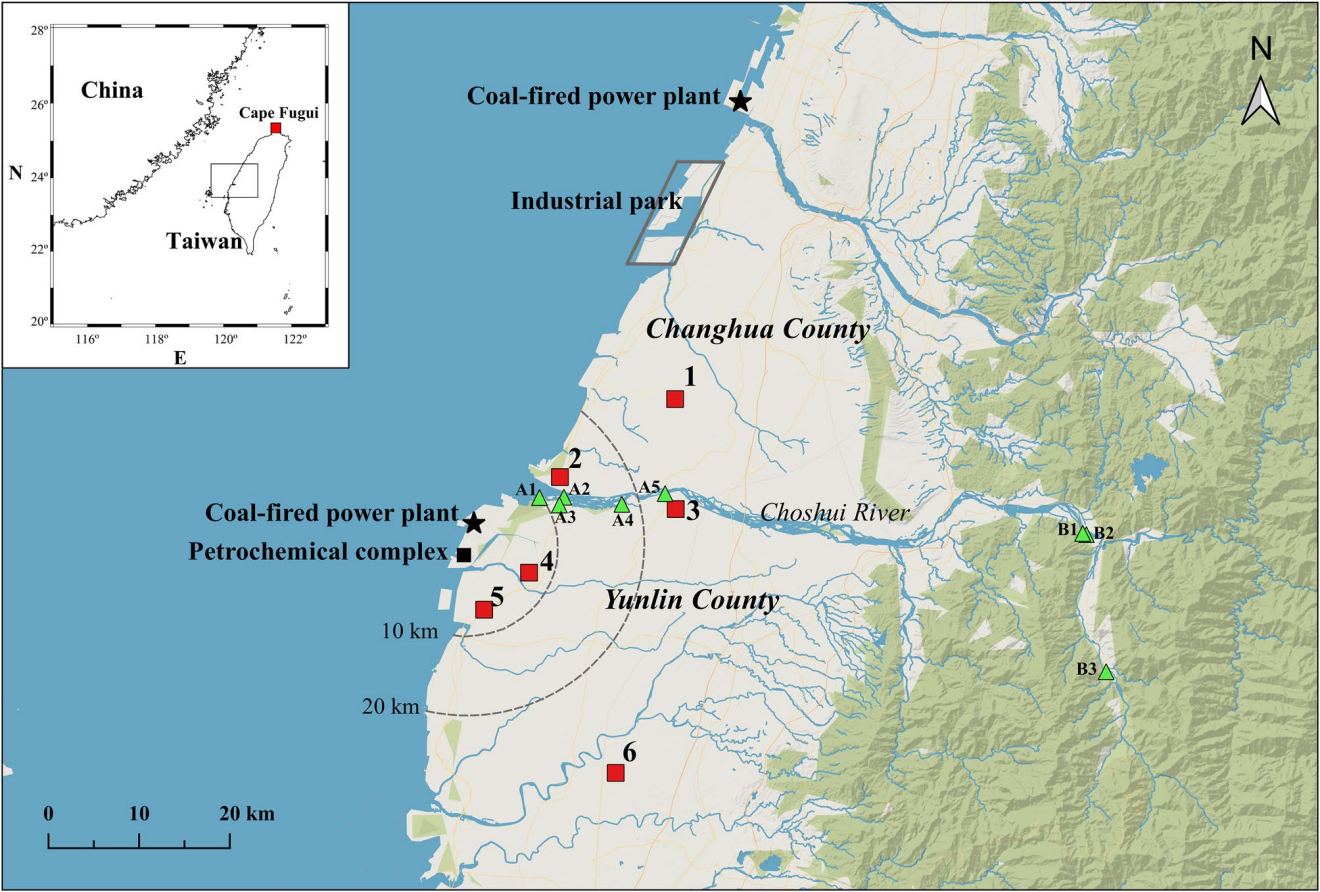
Map of the study area in Changhua and Yunlin Counties, central Taiwan. The red square represents the PM_10_ sampling site, and the green triangle represents the sediment sampling site.

Although some efforts have been made to study the Pb isotope ratios of PM in northern Taiwan^20,23^, the information available on Pb isotopic variations in PM_10_ remains limited. For instance, the constraints on local end-members and variations in isotopic signals over time and during different events have not been thoroughly studied. In the present study, PM_10_ was collected in central Taiwan during events of different types, including local events, an LRT event, and a dust storm (Fig. 1, and “Materials and methods” for the definition of each event type). Details about the sampling sites and period, wind speed and direction, temperature, precipitation, and relative humidity are summarized in Table 1. The main purposes of this study were, for the first time, (1) to characterize the chemical properties and Pb isotope ratios of local pollutants under different wind conditions (on daily basis) in central Taiwan, and (2) to identify the possible Pb source in PM_10_ during each event using the chemical characteristics and Pb isotope ratios.

**Table 1:** Table of sampling periods, sampling sites, and sampling parameters for each event.

Event type	Sampling periods	Sites	WD	WS (m/s)	T (ºC)	RH (%)	P (mm)
Local (moderate-wind-speed)	11/13–15 (2018)	#1–#6	North-Northwest	2.7–6.8	23.9–25.5	67–81	0
Local (low-wind-speed)	11/25 (2018)	#1–#6	Northwest, Southeast	0.95–3.0	17.9–22.5	75–98	1.5–8
Local (high-wind-speed)	12/7 (2018)	#1–#6	North	3.9–11.7	21.0–22.6	68–88	0
Long-range transport	10/4–5 (2019)	#1–#6,	Northeast-Northwest	0.93–1.2	26.2–27.4	82–90	0
	10/4–5 (2019)	Cape Fugui	East	4.0	25.8	79	0
Dust storm	10/30–31 (2019)	#5	North	6.5	24.3	77	0
Transition	10/31–11/1 (2019)	#5	North	5.4	25.1	80	0

WD: wind direction; WS: averaged wind speed; T: temperature; RH: relative humidity; P: precipitation. Meteorological data at each site is available on the EPA environmental resource database and Central Weather Bureau in Taiwan.

## Results and discussion

### PM_10_, ion, and metal concentrations

The variations of PM mass, metal and ion concentrations for PM_10_ collected from each event are summarized in Supplementary Table S1. Supplementary Fig. S1 illustrates variations in the daily average values of air quality parameters (including PM_10_, PM_2.5_, O_3_, and NO_x_/O_3_ ratio provided by the Taiwan Environmental Protection Administration [TEPA]) covering this study’s sampling periods (during the northeast monsoon in 2018 and 2019). All sampling campaigns were divided into local, LRT, and dust storm events based on the air mass back trajectory analysis, transport of nitric acid from outside Taiwan (reanalysis data), NO_x_/O_3_ ratio, and Al concentration in PM_10_. The local events were subdivided into low- (11/25, 2018), moderate- (11/13–15, 2018), and high-wind-speed (12/7, 2018) events based on wind speed criteria (see Materials and Methods for the details). The PM_10_ concentrations during all events in central Taiwan were high (Table 2), and most of them exceeded those stated in the Air Quality Guidelines provided by the World Health Organization (20 μg m^−3^ and 50 μg m^−3^ for annual mean and daily mean, respectively)^36^. In general, the PM_10_ concentration was relatively low (15.7–74.0 μg/m^3^) during the low-wind-speed event, while it reached the highest values during the high-wind-speed event (57.1–121 μg/m^3^). A moderate correlation (r = 0.59, p = 0.01) between PM_10_ and wind speed was found during the local events, suggesting that PM_10_ concentration in central Taiwan is most likely affected by factors related to the wind speed. During the low-wind-speed local event, elevated concentrations of water-soluble ionic species ([NO_3_^−^] = 8277–16,062 ng/m^3^, [NH_4_^+^] = 4258–8354 ng/m^3^, and [SO_4_^2−^] = 4794–9091 ng/m^3^) indicated that the atmospheric condition became stagnant as the wind weakened, and pollutants accumulated. Substantially lower concentrations of crustal elements (Al, Fe, and Ti) during this low-wind-speed event suggested a relatively small contribution from crustal materials. During the high-wind-speed local event, concentrations of ionic species decreased ([NO_3_^−^] = 2563–3127 ng/m^3^, [NH_4_^+^] = 182–1091 ng/m^3^, and [SO_4_^2−^] = 2661–4885 ng/m^3^) while concentrations of crustal elements increased (see Supplementary Table S1). During the LRT event (10/4–5, 2019), high concentrations of both PM_10_ (66.0–92.2 μg/m^3^) and water-soluble ionic species ([NO_3_^−^] = 10,665–24,430 ng/m^3^, [NH_4_^+^] = 9754–15,472 ng/m^3^, and [SO_4_^2−^] = 16,445–23,725 ng/m^3^) and heavy metals (V, Ni, As, Cd, and Pb) indicated worsening air quality caused by anthropogenic activities. By contrast, elevated PM_10_ (78.1 μg/m^3^) and Al concentration during the dust storm event (10/30–31, 2019) reflected greater contributions from crustal materials. To trace the source of PM_10_ in central Taiwan, we further applied enrichment factors (EFs), elemental ratios, and the Pb isotope data in PM_10_; this is discussed in the following sections.

**Table 2:** PM_10_ mass, sampling duration, filtered air volume, and selected elemental ratios for PM_10_ samples collected in this study.

Site	PM_10_ (μg/m^3^)	Sampling duration (h)	Volume (m^3^)	Fe/Al	V/Ni	Cd/Pb
**Local (moderate-wind-speed)**						
#1	108	10	689	2.18	1.62	0.026
#2	52.0	12	792	1.22	1.64	0.025
#3	62.0	28	1730	1.49	1.27	0.027
#4	116	12	781	1.31	1.91	0.023
#5	46.5	23	1457	1.72	1.65	0.031
#6	67.1	12	774	1.33	1.41	0.024
**Local (low-wind-speed)**						
#1	61.2	18	1241	2.16	1.47	0.038
#2	15.7	2	118	–	–	–
#3	78.1	18	1017	1.62	1.65	0.031
#4	59.5	18	1153	1.39	2.19	0.027
#5	53.1	18	1070	1.29	1.75	0.027
#6	48.4	18	1188	2.30	1.72	0.026
**Local (high-wind-speed)**						
#1	57.1	12	838	1.65	1.19	0.015
#2	42.2	12	792	1.81	1.59	0.020
#3	80.2	12	763	1.54	1.39	0.019
#4	116	12	829	1.22	2.33	0.016
#5	121	12	784	1.03	2.25	0.014
#6	66.1	12	792	2.66	1.58	0.030
**Long-range transport**						
#1	82.6	17	1132	0.81	2.28	0.053
#2	83.0	17	1130	0.78	2.42	0.054
#3	83.3	17	1122	0.79	2.31	0.061
#4	82.8	17	1193	0.90	2.29	0.057
#5	66.0	17	1122	0.74	2.48	0.054
#6	92.2	17	1122	0.58	2.29	0.051
Cape Fugui	17.9	17	1235	0.87	2.38	0.037
Dust storm	78.1	24	1584	0.71	2.02	0.031
Transition	59.0	24	1584	0.70	1.92	0.030
Sediments (A)				042–0.53	1.85–2.94	0.007–0.089
Sediments (B)				0.40–0.43	1.87–2.55	0.0009–0.0018
UCC^61^				0.44	3.00	0.005

### Enrichment factors and elemental ratios in PM_10_

**Figure 2:**
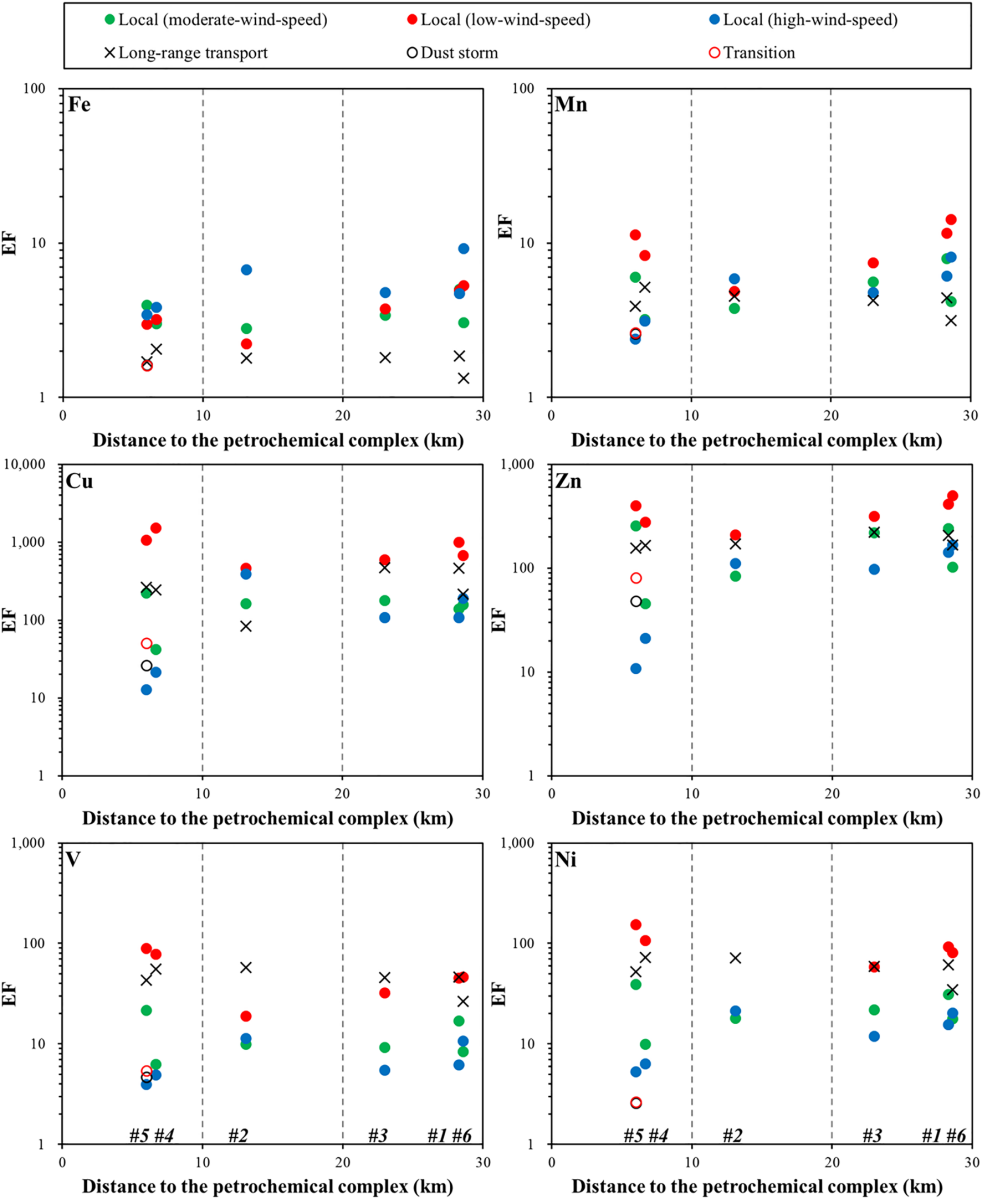

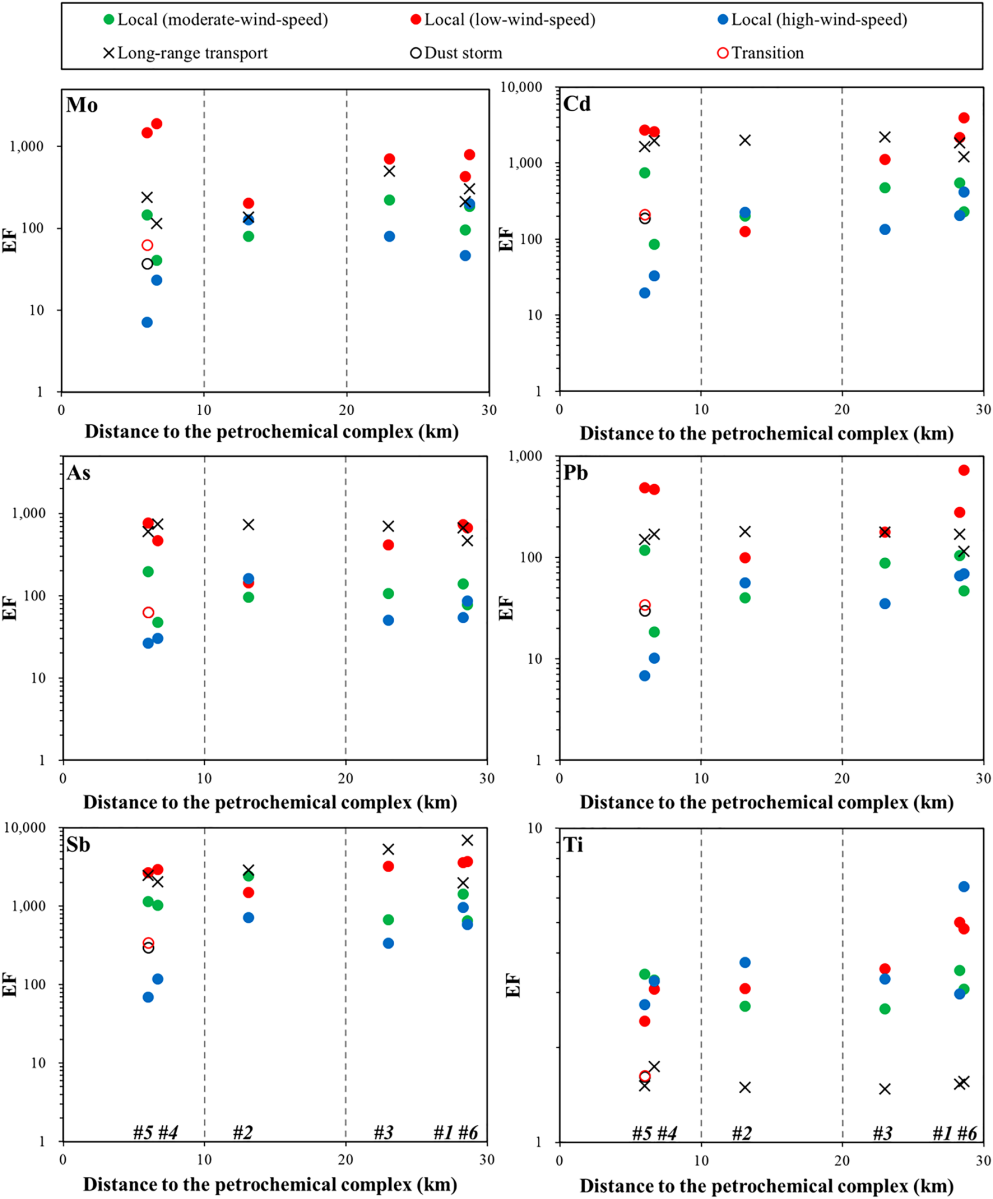
Enrichment factors (EFs) of elements (Fe, Mn, Cu, Zn, V, Ni, Mo, Cd, As, Pb, Sb, and Ti) versus distance to the petrochemical complex for all events collected in this study.

Elements, such as V and Ni, have been widely used as indicator elements of oil combustion^9,37^, and Mo is often regarded as a tracer for heavy oil combustion^38,39^. By investigating metal compositions of filterable stack total suspended particles emitted from industrial areas in Taiwan, Lin et al.^40^ reported that Mn, Zn, and Pb are critical marker elements for electric arc furnace steel plants, and that As, Cd, Sb, and Pb are potential indicator elements for coal-fired power plants. The EFs of these elements, therefore, can be further used to discuss anthropogenic sources. The EFs in PM_10_ at each study site were plotted against distance to a petrochemical complex to illustrate the distribution patterns of trace metals in this region. As depicted in Fig. 2, the EFs were high in the low-wind-speed local event, with the highest EFs of V (> 78), Ni (> 108), Cu (> 1056), and Mo (> 1478) at sites #4 and #5, the nearest sites to the petrochemical complex. Relative to the low-wind-speed event, the EFs decreased during the moderate-wind-speed event and reached the lowest values during the high-wind-speed local event. The EFs also varied significantly at sites, especially during the high-wind-speed local event, indicating that contributions from local sources changed at sites. During the LRT event, EFs of Fe and Ti had lower values than those observed during the local events, while high values were observed for most heavy metals. During the dust storm event, low EFs might have resulted from the dilution effect due to relatively higher contributions of crustal materials (Fig. 2).

To better assess the emission sources, elemental ratios such as Fe/Al, V/Ni, and Cd/Pb are often used as indicators of dust sources^41,42^, source of oil combustion^43–45^, and source of industrial activities^9,46^, respectively. The selected elemental ratios for PM_10_ collected in this study are summarized in Table 2. High Fe/Al ratios (1.03–2.66) were found during the local events, while low Fe/Al ratios (0.58–0.90) were found during the LRT event. The higher Fe/Al during the local events indicated Fe emission from steel plant or oil combustion^40,47,48^; the lower Fe/Al suggested PM might originate from different sources. Fe/Al ratio of 0.71 was observed during the dust storm event. This ratio is similar to those observed in a major Asian dust storm event^49^ and dust from Northern China^41^, indicating that PMs during the dust storm event were transported from Northern China.

A high correlation between V and Ni (r = 0.88, p < 0.001) was obtained for PM_10_ collected during the local events, suggesting the presence of one major V–Ni source. V/Ni ratio showed a narrow range between 1.19 and 2.33 during the local events, and slightly higher V/Ni ratios (2.28–2.48) were observed during the LRT event. According to previous studies, higher V/Ni ratios were reported in heavy oil combustion (V/Ni = 3–4)^43^ and ship emissions (V/Ni = 2.5–5)^44,45^, whereas lower V/Ni ratios might indicate additional Ni from other sources (especially oil combustion)^50^. Cd/Pb ratios varied between 0.014 and 0.038 during the local events. Chen et al.^24^ found a similar Cd/Pb ratio (0.033) for local emissions in central Taiwan, and a lower Cd/Pb ratio (0.024) was observed during high PM loading episodes. Hsu et al.^51^ also reported a Cd/Pb ratio of 0.030 for PM_10_ during summer in Taipei, and Cd/Pb decreased to 0.024 and 0.018 during the northeast monsoon and Asian dust periods, respectively. The Cd/Pb ratios found during the local events were similar to those obtained from anthropogenic activities in Europe (0.022)^52^. By contrast, high Cd/Pb ratios (0.051–0.061) were found during the LRT event, and these values were not observed in Taiwan before, indicating PM originated from non-local pollutants. The high Cd/Pb ratios were reported for PM in megacities in China^53^, these high ratios possibly indicated the origins of PM from non-ferrous metal production^52^.

**Figure 3:**
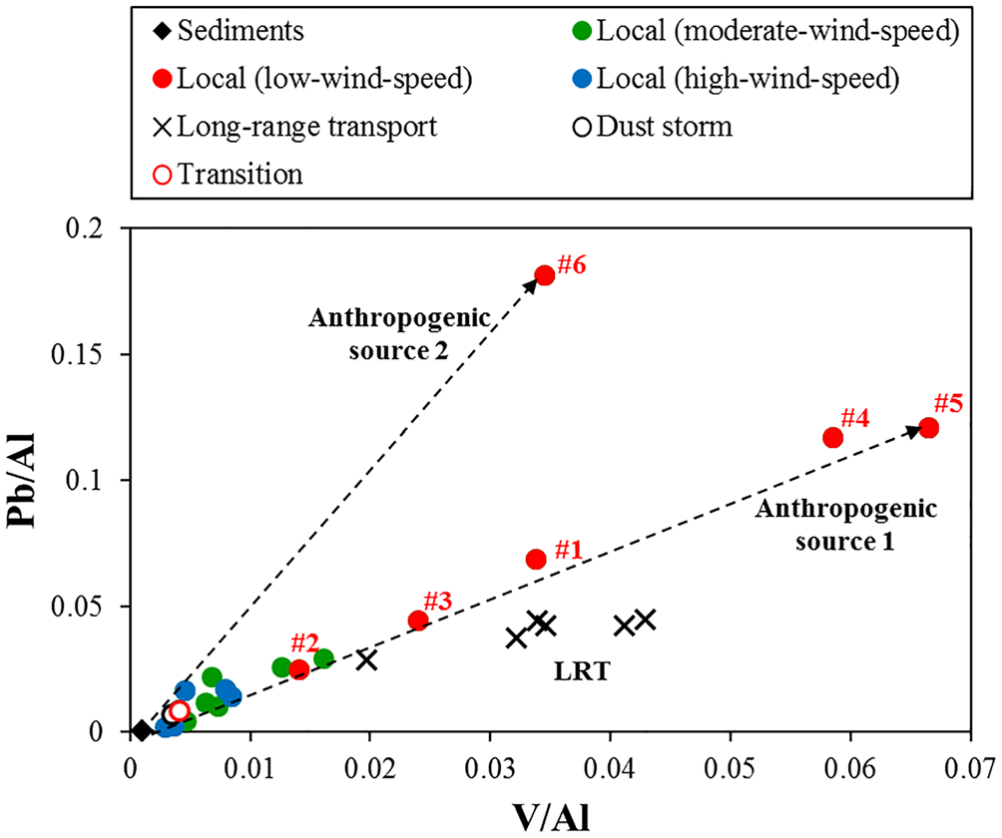
Plot of Pb/Al versus V/Al for PM_10_ and sediments collected in central Taiwan.

As mentioned above, Al, V, and Pb are marker elements for different sources; V/Al was plotted against Pb/Al in PM_10_ collected during the local events to illustrate sources contributed to these metals in central Taiwan. As shown in Fig. 3, at least three primary sources contributed to these metals in PM_10_ during the local events. Besides, an additional source was identified in this region during the LRT event. Multiple regression analysis was performed between PM_10_ and elemental Al, V, and Pb concentrations for PM_10_ collected during the local events. A high correlation (r = 0.89, p < 0.001) was obtained in this study, suggesting that the sources of PM_10_ in central Taiwan are possibly tracked by these chemical characteristics (including Pb) of PM_10_. We, therefore, further evaluate the potential of utilizing Pb isotope ratios of PM_10_ for tracing sources of Pb in PM_10_ in central Taiwan.

### Pb isotope compositions of potential PM_10_ sources in Taiwan

#### Anthropogenic sources

**Figure 4:**
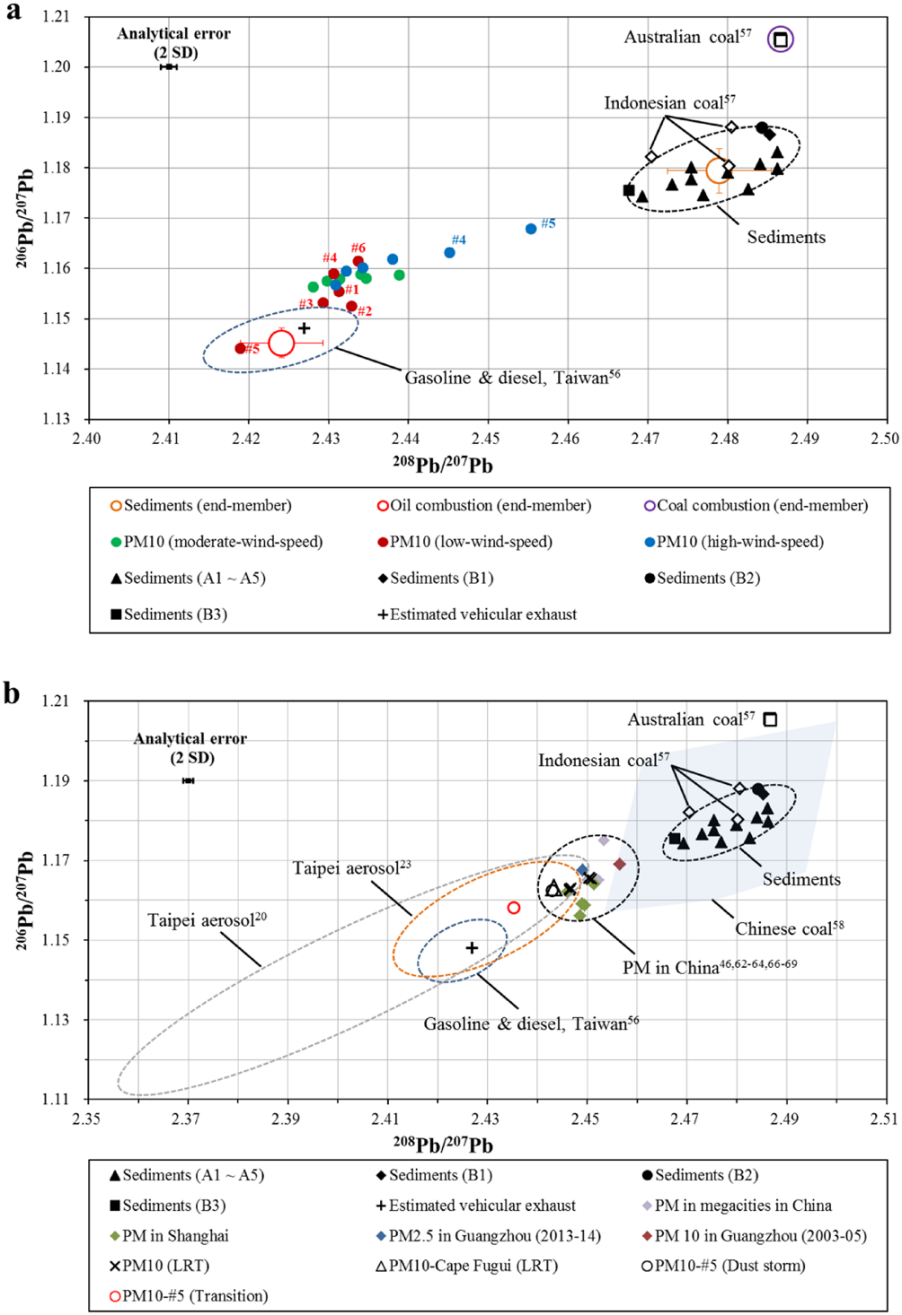
(**a**) Triple isotope plot of the Pb in PM_10_ during the local events. Pb isotope ratios of potential end-members (e.g., Oil combustion/refineries, coal combustion, and sediments) are also shown as red, purple, and orange open circles, respectively. (**b**) Triple isotope plot of the Pb in PM_10_ during the long-range transport and the dust storm events. Pb isotope ratios of PMs collected in Taipei^20,23^, China^46,62–64,66–69^, are also plotted for comparison.

The major anthropogenic sources of Pb are emissions from oil combustion (including oil boilers, vehicle exhaust, and oil refineries), coal combustion, and high-temperature industrial processes (e.g., steel plants)^54^. Taiwan is an island with limited energy resources and mainly relies on imported resources from other countries (up to 98%). According to Taiwan’s Bureau of Energy, oil, coal, and natural gas accounted for 48%, 29%, and 15%, respectively, of Taiwan’s total primary energy consumption in 2018^55^. Since 2012, Taiwan has been importing crude oil from Saudi Arabia (31%), Kuwait (21%), and others in similar proportions. Taiwan has been imported coal mainly from Australia (35–50%) and Indonesia (26–40%) since 2012. The unique Pb isotopic signatures of these sources facilitate the distinguishing of Pb sources. Yao et al.^56^ investigated the Pb isotope compositions of commercial oils from two main oil product suppliers in Taiwan, with a ^206^Pb/^207^Pb ratio of 1.141 to 1.151 and a ^208^Pb/^207^Pb ratio of 2.417 to 2.429 for unleaded gasoline, and a ^206^Pb/^207^Pb ratio of 1.148 to 1.149 and a ^208^Pb/^207^Pb ratio of 2.431 to 2.433 for diesel; they also estimated the Pb isotope ratios of vehicular emissions in Taiwan to be 1.148 for ^206^Pb/^207^Pb and 2.427 for ^208^Pb/^207^Pb based on the sales and market share of gasoline and diesel in Taiwan (Fig. 4). Díaz-Somoano et al*.*^57^ reported that Australian coal and Indonesian coal have high Pb isotope ratios (with ^206^Pb/^207^Pb = 1.205 to 1.211 and ^208^Pb/^207^Pb = 2.487, and with ^206^Pb/^207^Pb = 1.180 to 1.188 and ^208^Pb/^207^Pb = 2.470 to 2.481, respectively); Bi et al.^58^ also reported high value of Pb isotopic ratios for Chinese coals in different regions (^206^Pb/^207^Pb = 1.161 to 1.223, with an average value of 1.188, and ^208^Pb/^207^Pb = 2.453 to 2.511, with an average value of 2.482), as illustrated in Fig. 4. Regarding Pb emissions in high-temperature industries, no Pb isotope data are available for Taiwan; therefore, constraining Pb isotope ratios for these emissions in the future is needed.

#### Natural source

Studies have demonstrated that, under high wind speeds, windblown dust influences the air quality in the downstream segment of the Choshui River, particularly during the northeast monsoon season^27,59,60^. However, these studies have not provided a quantitative estimation of the contribution of local dust to PM_10_ in central Taiwan. Recently, Hsu et al.^25^ attempted to estimate this contribution by using a receptor-based source apportionment model. Herein, we provide an alternative method for calculating the contribution based on the Pb isotope ratios of PM_10_ and potential end-members. To constrain the isotopic signals of natural materials and further estimate their contribution to Pb in PM_10_, we analyzed sediments from the Choshui River catchment (Fig. 1) for their metal concentrations and Pb isotope ratios. Because sediments in downstream areas could be influenced by human activities, sampling was conducted in both the downstream (group A) and upstream (group B) segments of the Choshui River catchment (Fig. 1). The EFs of these sediments were calculated relative to the upper continental crust^61^, and the results are presented in Supplementary Fig. S2. Although the EFs of Cd were higher (EF_Cd_ = 9.9 ± 8.6) downstream than upstream (EF_Cd_ = 0.5 ± 0.2), most EF values were close to unity for sediments collected from the upstream and downstream segments of the Choshui River catchment. This indicates that the element contents of this river’s sediments are similar to the average composition in the upper continental crust. All the sediments analyzed in this study exhibited higher Pb isotopic ratios (^206^Pb/^207^Pb = 1.174–1.188; ^208^Pb/^207^Pb = 2.468–2.486, Supplementary Table S3) compared with aerosols collected in central Taiwan during this study (Fig. 4).

### Pb source of PM_10_ in central Taiwan: local events

As shown in the triple Pb isotope plot (Fig. 4a), PM_10_ during the low-wind-speed event (11/25, 2018) had the lowest Pb isotope ratios, with a ^206^Pb/^207^Pb ratio of 1.144–1.161 and a ^208^Pb/^207^Pb ratio of 2.419–2.434 (Table 3). These ratios were close to or within the range of those reported for gasoline and diesel in Taiwan, suggesting that oil combustion/refineries are the main sources of Pb during the low-wind-speed conditions. Under this low-wind-speed condition, PM_10_ at sites #4 and #6 exhibited slightly elevated Pb isotope ratios, indicating the presence of Pb sources other than oil combustion (Fig. 4a). For instance, site #6, the southernmost site in the study region, exhibited high Pb isotope ratios (^206^Pb/^207^Pb = 1.161 and ^208^Pb/^207^Pb = 2.434) and a high ratio of Pb/Al (Figs. 3 and 4a). From a combination of geochemical constraints (i.e. higher Pb/Al and Pb isotope ratios), we inferred that the elevated Pb isotope ratios at sites #4 and #6 were the result of elevated contributions from coal-fired power plants and steel plants. Pb isotope emission signals from steel plants, however, are still required to further assess the contribution from such plants.

**Table 3:** Pb concentration and Pb isotope ratios of PM_10_ samples collected during each event type in central Taiwan.

Site	^206^Pb/^204^Pb	2 se	^206^Pb/^207^Pb	2se	^208^Pb/^207^Pb	2 se	Pb conc. (ng/m^3^)
**Local (moderate-wind-speed)**							
#1	18.0368	0.0018	1.1576	0.00018	2.4298	0.00041	22.9
#2	18.0577	0.0019	1.1580	0.00019	2.4347	0.00044	5.63
#3	18.0154	0.0021	1.1564	0.00021	2.4281	0.00052	18.2
#4	18.0757	0.0016	1.1586	0.00015	2.4388	0.00033	7.80
#5	18.0399	0.0024	1.1579	0.00023	2.4314	0.00053	10.8
#6	18.066	0.0021	1.1588	0.00021	2.4340	0.00050	8.97
**Local (low-wind-speed)**							
#1	17.9996	0.0021	1.1554	0.00020	2.4313	0.00047	11.2
#2	17.9858	0.0016	1.1525	0.00015	2.4329	0.00032	1.33
#3	17.9705	0.0021	1.1531	0.00021	2.4293	0.00048	15.6
#4	18.0487	0.0028	1.1589	0.00028	2.4307	0.00065	25.7
#5	17.7884	0.0029	1.1441	0.00027	2.4189	0.00063	18.7
#6	18.1116	0.0018	1.1614	0.00018	2.4337	0.00046	30.5
**Local (high-wind-speed)**							
#1	18.0192	0.0028	1.1568	0.00026	2.4309	0.00058	18.2
#2	18.1095	0.0018	1.1618	0.00017	2.4380	0.00040	5.45
#3	18.0704	0.0017	1.1594	0.00016	2.4322	0.00034	15.6
#4	18.1417	0.0014	1.1631	0.00013	2.4452	0.00031	7.89
#5	18.2533	0.0016	1.1679	0.00015	2.4554	0.00032	6.19
#6	18.0924	0.0024	1.1601	0.00024	2.4343	0.00051	12.7
**Long-range transport**							
#1	18.2004	0.0012	1.1631	0.00011	2.4468	0.00025	27.4
#2	18.2451	0.0014	1.1653	0.00013	2.4508	0.00029	28.1
#3	18.1876	0.0014	1.1626	0.00012	2.4464	0.00027	23.3
#4	18.2438	0.0011	1.1656	0.00010	2.4504	0.00022	26.4
#5	18.2442	0.0012	1.1655	0.00011	2.4503	0.00025	24.4
#6	18.1864	0.0014	1.1629	0.00014	2.4465	0.00034	30.3
Cape Fugui	18.1982	0.0011	1.1632	0.00011	2.4433	0.00024	4.91
Dust storm	18.1658	0.0011	1.1625	0.00010	2.4429	0.00025	17.3
Transition	18.0900	0.0012	1.1582	0.00011	2.4352	0.00024	12.4

During the moderate- (11/13–15, 2018) and high-wind-speed (12/7, 2018) events, PM_10_ had higher Pb isotope ratios compared with PM_10_ observed during the low-wind-speed event, with a ^206^Pb/^207^Pb ratio of 1.157–1.168 and a ^208^Pb/^207^Pb ratio of 2.431–2.455 (Table 3, Fig. 4a). The higher Pb isotope ratios suggested that Pb originated from either sediments or coal combustion because of the overlap of ratios between Indonesian coal and local sediments. This overlap of isotopic signals may hamper the explanation of the source. However, these two potential sources can be differentiated using EF values. As illustrated in Figs. 2 and 4a, PM_10_ at sites #4 and #5 during the high-wind-speed event had the highest Pb isotope ratios and the lowest EF of Pb (< 10), indicating that these high ratios were mainly contributed by sediments rather than by other sources. The high concentrations of Al (3107 and 3656 ng/m^3^, respectively) in PM_10_ at these two sites also support the increased contribution of crustal materials during the high-wind-speed event, confirming that natural dust is the dominant source of PM_10_ in central Taiwan during the northeast monsoon season.

In summary, PM_10_ in central Taiwan was predominantly attributed to oil combustion processes (i.e., industrial activities from the petrochemical complex and vehicular emissions), whereas the signals from coal-fired power and steel plants varied between sites. When the wind speed increased, another crucial PM_10_ source in central Taiwan was local sediments.

### Pb source of PM_10_ in central Taiwan: LRT and dust storm events

During October 2–4, 2019, Taiwan’s main island suffered severe air pollution because of LRT of pollutants from China and poor diffusion conditions. The air mass back trajectory revealed that the air parcels possibly originated from Southeast China, passing the Pearl River Delta (PRD) region, a major industrial and economic center (Supplementary Fig. S4). The European Centre for Medium-Range Weather Forecasts (ECMWF) Atmospheric Composition Reanalysis 4 (EAC4) reanalysis dataset reveals that a nitric acid plume formed in Southeast China and gradually moved northeastward to Taiwan (Supplementary Fig. S5). In this study, PM_10_ samples were collected on October 4, 2019. As discussed previously, the high concentrations of ionic species (sulfate, nitrate, and ammonium) and heavy metals (V, Ni, As, Cd, and Pb) indicated that the air quality became increasingly polluted (Supplementary Table S1). PM_10_ collected during this period provided more radiogenic results compared with those during local events, with a ^206^Pb/^207^Pb ratio of 1.163–1.166 and a ^208^Pb/^207^Pb ratio of 2.446–2.451 (Table 3; Fig. 4b).

High Pb isotope ratios indicated that contaminants may have originated from China because such ratios in aerosol samples have commonly been observed there. An earlier study investigated the Pb isotope ratios in airborne particles in urban and suburban areas of Guangzhou^62^, revealing average Pb isotope ratios during winter of 1.1631 ± 0.0058 and 2.4579 ± 0.0081 for ^206^Pb/^207^Pb and ^208^Pb/^207^Pb, respectively (Fig. 4b). More recently, similar Pb isotope ratios (^206^Pb/^207^Pb = 1.1675 ± 0.0040 and ^208^Pb/^207^Pb = 2.4491 ± 0.0066) were observed in PM_2.5_ in Guangzhou, suggesting that such ratios in PM have remained high in the PRD^63^. According to relevant studies, coal combustion became the major source of Pb in the atmosphere after the phasing-out of leaded gasoline, leading to more radiogenic isotope ratios being observed for particles in the atmosphere in China^64,65^. High Pb isotopic ratios were also reported for aerosols in areas with high levels of industrial emissions^46,66–69^ (Fig. 4b). Thus, the high Pb isotope ratios in our PM_10_ samples collected during the LRT event along with low Fe/Al and high Cd/Pb ratios suggested that the pollutants were most likely transported from China to Taiwan; furthermore, these signals were not observed during the local events. During the LRT event, PM_10_ collected at Cape Fugui (a regional background site for monitoring LRT from outside Taiwan located at the northern tip of Taiwan) also exhibited similar Pb isotope ratios (^206^Pb/^207^Pb = 1.163 and ^208^Pb/^207^Pb = 2.443, Fig. 4b).

During the dust storm event (10/30–31, 2019), the air mass back trajectory revealed that the air parcels primarily originated from Northern China and passed through Shanghai, a megacity and economic center in East China, before reaching Taiwan (Supplementary Fig. S4). The dust-mixing ratio estimated by the EAC4 reanalysis indicated that the dust storm was mainly derived from Northern China, with the dust plume gradually being transported to East China (Supplementary Fig. S6). When the dust storm passed through Shanghai, a nitric acid plume derived from East China gradually moved southward to the Taiwan Strait (Supplementary Fig. S7). During this dust event, PM_10_ samples were collected for 2 days consecutively at site #5. The results indicated that PM_10_ had higher Pb isotope ratios on the first day of sampling, with ^206^Pb/^207^Pb = 1.163 and ^208^Pb/^207^Pb = 2.443, whereas the Pb isotope ratios rapidly changed to lower values on the second day, with ^206^Pb/^207^Pb = 1.158 and ^208^Pb/^207^Pb = 2.435 (Fig. 4b). The EFs of most metals in the PM_10_ collected during the dust storm event were low (< 343, Fig. 2), indicating that the PM_10_ was a mixture of pollutants and natural dust. This was further supported by the elevated Al concentrations (2324 ng/m^3^) in the collected samples. The Pb isotope ratios of PM_10_ on the first day reflected that this dust storm collected pollutants in East China and then transported them to Taiwan, as depicted by the reanalysis dataset and the higher Pb isotope ratios found in PM from East China. On the second day of sampling during the dust event (defined as Transition), the Pb isotope ratios of PM_10_ decreased toward those detected during the local events, suggesting that the contribution from local emissions (oil combustion/refineries) became dominant. This again confirmed that oil combustion and oil refineries (characterized by low Pb isotopic compositions) are the major sources of Pb in PM_10_ in central Taiwan.

### Estimating the relative contribution to Pb in PM_10_

Because distinctive Pb isotope ratios were found for each end-member, we were able to estimate their relative contributions to Pb in PM_10_. We adopted a ternary mixing model to calculate the relative contributions to Pb in PM_10_ using the following equations:

1 $${R}_{PM10}={{\sum }_{i=1}^{3}{f}_{i}{ R}_{i}}$$

2 $${{\sum }_{i=1}^{3}{f}_{i}=1}$$

where *R*_*PM*10_ is the observed ^206^Pb/^207^Pb (or ^208^Pb/^207^Pb) ratio of PM_10_, *Ri* is the assumed ^206^Pb/^207^Pb (or ^208^Pb/^207^Pb) ratio for each end-member, and *fi* is the fraction of the contribution of each end-member.

The end-members discussed in this study (i.e., oil combustion/refineries, coal combustion, and sediments) were assigned accordingly, as shown in Fig. 4a. We assumed that the end-member oil combustion/refineries were the average isotopic signatures of the oil products (gasoline and diesel, ^206^Pb/^207^Pb = 1.146 and ^208^Pb/^207^Pb = 2.425) investigated in Taiwan and assumed that the end-member coal combustion was the average isotopic signature of Australian coal (^206^Pb/^207^Pb = 1.206 and ^208^Pb/^207^Pb = 2.487) since Australian coal occupied half of the coal imported into Taiwan. The Pb isotope ratios for the mixture of Australian coal and Indonesian coal (by import proportion) are also very close to those of Australian coal; therefore, we simplified the calculation by using Australian coal as the end-member. We also assumed that the end-member natural material was the average isotopic signature of sediments (^206^Pb/^207^Pb = 1.179 and ^208^Pb/^207^Pb = 2.479) collected in this study.

**Figure 5:**
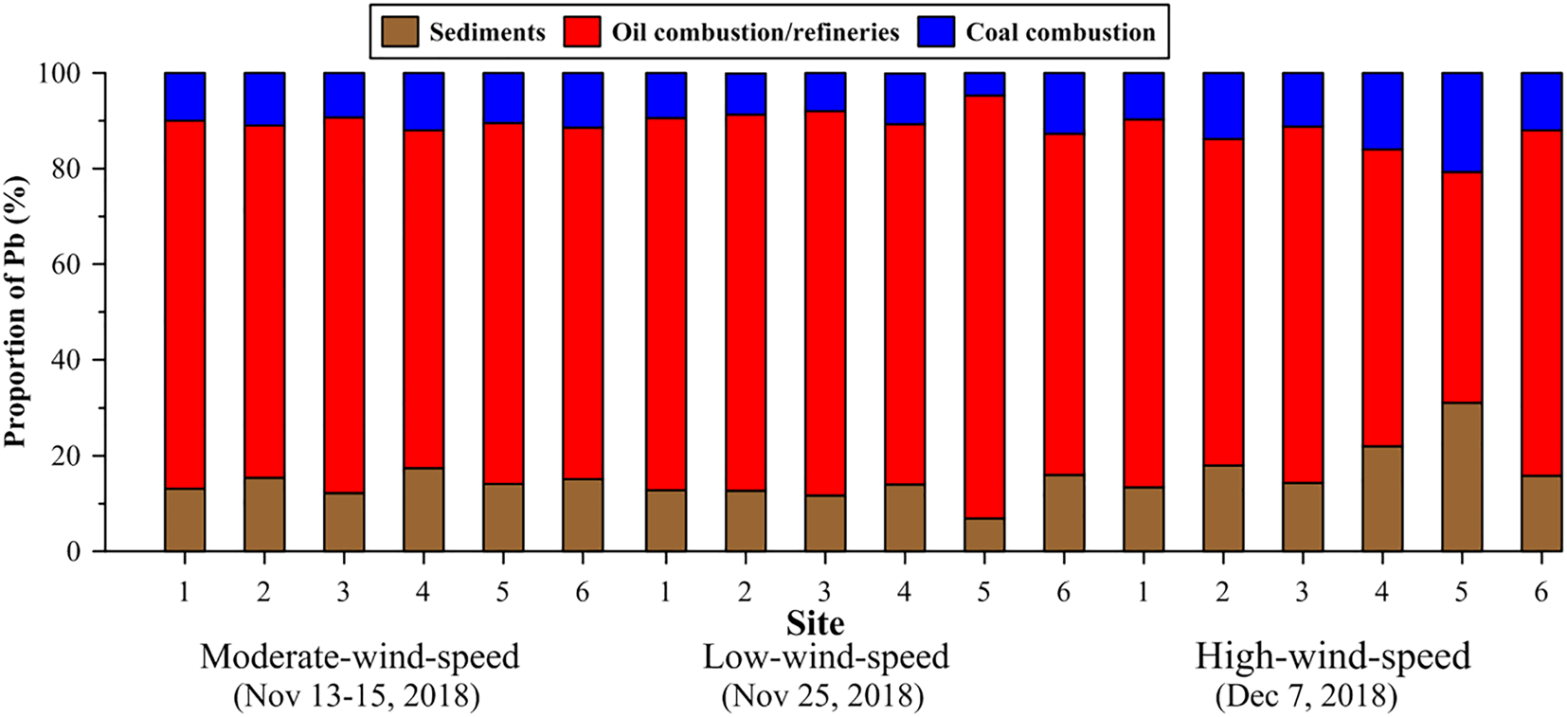
MixSIAR model outputs of the relative contributions (mean value, with the upper and lower bounds being the 2.5% and 97.5% credible intervals, see Supplementary Table S2) from each end-member to Pb in PM_10_ at each site during the local events.

A Bayesian mixing model, MixSIAR^70,71^, was employed to calculate each end-member contribution to Pb in PM_10_. This model incorporates the uncertainties of the isotopic signatures for each source, and it was successfully applied to assess Pb sources in isotopic mixtures quantitatively^72^. The model results revealed the contribution from oil combustion to be predominant (48–88%) during the local events, whereas coal combustion made a minor contribution (< 21%) in central Taiwan (Supplementary Table S2, Fig. 5). On the other hand, contributions from sediments increased significantly from 13 to 31% during the high-wind-speed event and were highly variable among sites. The high proportions of oil combustion suggested an approach for mitigating emissions in the future.

During the LRT and dust storm events, the Pb isotope ratios of PM_10_ might have been overprinted by pollutants derived from China (as shown in Fig. 4b), and further complicated the contribution estimations. In such cases, Pb in PM_10_ was assumed to be dominated by the LRT of pollutants, and the approach presented here should be able to delineate the PM_10_ sources if the primary sources of Pb can be better constrained.

## Concluding remarks

This study investigated the chemical characteristics and sources of PM_10_ during various events of the 2018/2019 northeast monsoon season in central Taiwan. High concentrations of ionic species were found during the low-wind-speed local event, suggesting that local pollutants accumulated when the atmospheric condition stagnated. By contrast, high concentrations of crustal elements (e.g., Al, Fe, and Ti) were found during the high-wind-speed local event, suggesting the enhanced influence of local sediments. By employing EFs, elemental ratios, and Pb isotope ratios, we revealed an evident variation in the contributions from local sources. For instance, oil combustion was enriched in V and Ni and had the lowest Pb isotope ratios. Coal combustion was enriched in As, Sb, and Pb and had the highest Pb isotope ratios. Sediments were characterized by the unity of EFs and moderate Pb isotope ratios.

This study also included a ternary mixing model of Pb isotopes for the source apportionment of Pb in PM_10_ in central Taiwan. The contribution of Pb was dominated by oil combustion/refineries during local events (48–88%), whereas the contributions from coal combustion were lower (< 21%). The contributions from sediments were increased from 13 to 31% when the wind speed became high. All of the results were supported by the chemical characteristics of PM_10_ and the reanalysis dataset. Furthermore, a strong correlation (r = 0.89, *p* < 0.001, multiple regression analysis) between PM_10_ mass and the concentrations of Pb, V, and Al was observed in the study area, suggesting that the sources of PM_10_ in central Taiwan can be possibly tracked by using these chemical characteristics of PM_10_. By combining Pb isotope ratios, elemental ratios, EFs, and the reanalysis dataset, this study improved the constraints of PM_10_ sources during different events. Moreover, we demonstrated a multi-tracer approach to understand transport and the contributions of Pb in PM_10_ from various sources, which serves as a powerful tool for delineating complex atmospheres impacted by complex emission sources.

## Materials and methods

### Sampling site and PM_10_ collection

PM_10_ sampling was conducted at six sites located in the rural-fringe areas of Changhua, Yunlin, and Chiayi Counties in central Taiwan (Fig. 1), where the air quality is reported to be among the worst in Taiwan^73^. Several obvious anthropogenic sources of PM exist, such as a coal-fired power plant (one of the largest in the world) in Taichung City, Changhua coastal industrial park in Changhua County, and a petrochemical complex (including a coal-fired power plant) in Yunlin County. Moreover, sediments in the Choshui River catchment are the major source of PM derived from natural materials. Details about the sampling sites, sampling period, wind speed, wind direction, temperature, precipitation, and relative humidity are summarized in Table 1. A total of five sampling campaigns were conducted during the northeast monsoon season to collect PM_10_ samples under different types of events, including three local events in 2018, one LRT event, and one dust storm event in 2019. Each event type was defined based on meteorological data, announcements provided by the TEPA, air mass back trajectory analysis (Hybrid Single Particle Lagrangian Integrated Trajectory [HYSPLIT]), the global reanalysis dataset (EAC4), NO_x_/O_3_ ratio, and Al concentration in PM_10_. The LRT event was defined as having air masses originating from potential high emission regions outside Taiwan (e.g., megacities in the coastal regions of China, see Supplementary Figs. S3 and S4), apparent transport of nitric acid to Taiwan (Supplementary Fig. S5), and a low ratio of NO_x_/O_3_ (see Supplementary Fig. S1). The low NO_x_/O_3_ ratio reflected that the air mass could have been transported from long distances, as NO_x_ has a shorter lifetime than O_3_ in the atmosphere^74^, and NO_x_ gets reacted faster than O_3_ during long-range transport. In contrast to the LRT event, a local event did not have the conditions mentioned above. A dust storm event was defined as air masses originating from Northern China (Supplementary Fig. S4), southeastward transportation of dust (Supplementary Fig. S6), and Al concentration > 1400 ng/m^3^ in PM_10_^75^. The local events were further divided into low-, moderate-, and high-wind-speed events based on the Beaufort wind scale^76^ of < 3.3 m/s (calm, light air, and light breeze), 3.3–8 m/s (gentle breeze, moderate breeze), and > 8 m/s (fresh breeze, strong breeze), respectively. Note that the PM_10_ samples were only collected at site #5 during the dust storm event because only one sampler was available.

PM_10_ samples were collected following the TEPA PM_10_ sampling protocol (NIEA A208). In brief, PM_10_ samples were collected at a flow rate of 66 ± 3 m^3^ h^−1^ on a polytetrafluoroethylene (PTFE) filter using a high-volume sampler (Tisch Environmental., Cleves, OH, USA) with a size-selective inlet (10 μm) attached on the rooftop (approximately 10 m above ground level). The sampling was conducted for 8–28 h, except for site #2 (2 h) on Nov 25, 2018. After collection, the filter was transferred into a plastic envelope and delivered to the conditioning room within a few hours. Furthermore, a total of 13 sediment samples were collected from eight sites (Fig. 1) to constrain the chemical and isotope signals of the natural source. The sediment samples were collected in both upstream and downstream segments of the Choshui River catchment with plastic bags, dried at 45 °C in an oven, ground and sieved through a 75 μm sieve, and stored in a desiccator.

### Chemical analysis

The PTFE filter was weighted for particle mass concentration with a microbalance after equilibration at 25 ± 1.5 °C under a relative humidity of 40% ± 5% for 24 h. Next, 1/9 of the filter was cut using a ceramic cutter, and the aliquot was then digested with an acid mixture of 9 mL of concentrated HNO_3_, 3 mL of concentrated HCl, and 3 mL of concentrated HF using a microwave (CEM Corp., Matthews, NC, USA). A two-stage heating procedure was employed; first, the mixture was heated to 170 °C over 20 min and maintained at this temperature for 10 min, followed by another round of heating to 200 °C over 7 min and maintaining for 10 min. After cooling, the solution was transferred into a PFA beaker and evaporated. The dried sample was re-dissolved with an acid mixture of 2 mL of concentrated HNO_3_ and 1 mL of concentrated HCl, and finally diluted to 50 mL with Milli-Q water. Ultrapure concentrated acids and Milli-Q water were used for sample preparation in this study. For sediment samples, 100 mg of sediment was weighed and digested following the aforementioned procedure. A total of 23 elements, including major and trace metal concentrations, were analyzed by using Q-ICP-MS (Agilent 7700X) with internal standards (Sc, Y, Rh, Tb, Lu, and Bi) to monitor the instrumental drift and matrix effect (NIEA M105); the analytical precision was better than 10% (RSD). The method detection limit for each element was determined by field blanks, as presented in Supplementary Table S4. For each batch of the sample digestion, the accuracy of metal analysis was examined according to two international standards, NIST SRM 1648a (urban PM) and NIES No. 30 (Gobi Kosa dust). The results of metal concentrations and recoveries for NIST SRM 1648a and NIES No. 30 are summarized in Supplementary Tables S5 and S6, respectively.

For the ion concentration, a sample aliquot of the filter was extracted with 50 mL of Milli-Q water in an ultrasonic bath for 30 min, followed by settling for 30 min. The solution was then filtered through a 0.45 μm PVDF filter (Merck KGaA, Darmstadt, Germany). Ion concentrations (SO_4_^2−^, NO_3_^−^, NH_4_^+^, and Cl^−^) were measured using ion chromatography (Thermo-Fisher Scientific, Waltham, MA, USA) with analytical precision better than 10% (RSD).

### Pb isotope analysis

An aliquot of the digested sample was transferred into an acid-cleaned PFA beaker. This solution was evaporated and re-dissolved with 2 mL of 2 M HNO_3_ and 0.07 M HF. The Pb fraction was extracted using *Sr-spec* ion exchange resin (Eichrom Technologies Inc., Lisle, IL, USA) following the steps modified from Pin et al.^77^. An international reference material (NIST 1648a) was also processed for each purification batch to assess the column chemistry performance. Pb was purified under a Class-10 laminar flow bench in a Class-10,000 clean room. The total procedural blanks for Pb were < 80 pg. Pb isotope ratios were measured using HR-MC-ICP-MS (Neptune PLUS, Thermo-Fisher Scientific) at the Institute of Earth Sciences, Academia Sinica (AS-IES). Standards and samples were doped with thallium (Tl) (NIST 997; ^203^Tl/^205^Tl ratio of 0.418673) to correct for instrumental mass fractionation^78^. Two standard reference materials (NIST SRM 981 and NIST SRM 1648a) were analyzed to assess the accuracy and long-term precision of the analytical protocol developed at AS-IES. The measured Pb isotope ratios for these international reference materials were in good agreement with the recommended values and are listed in Supplementary Table S7. The analytical uncertainties (2SD) for ^206^Pb/^207^Pb and ^208^Pb/^207^Pb were ± 0.0002 (n = 69) and ± 0.0003 (n = 69), respectively. Pb isotope ratios were reported as ^206^Pb/^207^Pb and ^208^Pb/^207^Pb in this study.

### Enrichment factor

The EF has been widely used to examine the contributions from natural and anthropogenic sources in aerosols^79–81^. The EF of elements was calculated using Eq. (3):

3 $$\text{EF}=\frac{{({X}_{i}/Al)}_{PM}}{{({X}_{i}/Al)}_{Crust}}$$

where (Xi/Al)_PM_ is the concentration ratio of element Xi to Al in PM, and (Xi/Al)_Crust_ is the concentration ratio of element Xi to Al in the upper continental crust^61^. In general, EF values close to unity indicate the predominance of crustal sources; EF values ≥ 5 indicate a significant contribution from noncrustal sources; and EF values higher than 10 indicate essentially anthropogenic origins^24,80,82^.

### Reanalysis datasets

The EAC4 global reanalysis dataset provided by the Copernicus Atmosphere Monitoring Service (CAMS) was applied to provide additional constraints on the sources and transportation of pollutants in this study. EAC4 reanalysis combines model data with global observations into a globally complete and consistent dataset; the dataset used in this study was generated using CAMS information (2020)^83^. The spatial resolution of the dataset was 0.75° latitude by 0.75° longitude, with a temporal resolution of 3 h.

In addition, air mass back trajectory analysis was used to track the origins of the air parcels transported to the study sites. Back trajectories were calculated using the HYSPLIT model maintained by the US National Oceanographic and Atmospheric Administration with a spatial resolution of 1° latitude by 1° longitude in the meteorological dataset^84^.

## Supplementary Information

Supplementary Information.
