# Supplementary material for: Tracing local sources and long-range transport of PM10 in central Taiwan by using chemical characteristics and Pb isotope ratios
Source: Sci Rep. 2021 Apr 7;11:7593. doi: 10.1038/s41598-021-87051-y (PMC8026966; doi:10.1038/s41598-021-87051-y)
Supplement: Supplementary file 1 — Supplementary Information. [file 41598_2021_87051_MOESM1_ESM.pdf]

Supplementary Information

Tracing local sources and long-range transport of PM_{10} in central Taiwan by using chemical characteristics and Pb isotope ratios

Po-Chao Wu[123] and Kuo-Fang Huang[2,*]

[1]Earth System Science Program, Taiwan International Graduate Program (TIGP), Academia Sinica, Taipei, Taiwan.
[2]Institute of Earth Sciences, Academia Sinica, Taipei, Taiwan.
[3]College of Earth Science, National Central University, Taoyuan, Taiwan.

***Corresponding author:**
Dr. Kuo-Fang Huang
Institute of Earth Sciences, Academia Sinica
128, Section 2, Academia Road, Nangang, Taipei 11529, Taiwan
Tel: +886-2-2783-9910 ext. 1616
Fax: +886-2-2783-9871
E-mail: kfhuang@earth.sinica.edu.tw

Table S1 Concentrations of mass (μg/m^3), major ions (ng/m^3) and metals (ng/m^3) of PM_{10} collected during each event.

**Sampling Date**	**11/13-15 (2018)**	**11/25 (2018)**	**12/7 (2018)**	**10/4-5 (2019)**		**10/30-31 (2019)**	**10/31-11/1 (2019)**
**Sites**	#1 – #6	#1, #3– #6	#1 – #6	#1 – #6	Cape Fugui	#5	#5
**PM_{10}**	46.5 – 116	48.4 – 74.0	42.2 – 121	66.0 – 92.2	17.9	78.1	59.0
**SO_4^{2-}**	4,569 – 11,496	4,794 – 9,019	2,661 – 4,885	16,445 – 23,725	3,942	7,336	6,343
**NO_3^-**	3,671 – 11,097	8,277 – 16,062	2,563 – 3,127	10,665 – 24,430	1,176	8,825	7,918
**NH_4^+**	1,096 – 4,791	4,258 – 8,354	482 – 1,091	9,754 – 15,472	1,102	2,688	1,994
**Cl^-**	1,789 – 7,057	861 – 2,872	2,377 – 6,666	60.0 –751	1,061	3,709	5,044
**Na**	1,385 – 4,243	790 – 1,370	1,510 – 4,061	508 – 723	1,301	3,613	4,854
**Mg**	194 – 394	81.4 – 167	130 – 740	126 – 162	171	1,023	854
**Al**	368 – 1,707	155 – 351	390 – 3,656	526 – 1,055	111	2,324	1,454
**K**	458 – 1,115	844 – 1,293	534 – 2,030	512 – 674	149	1,125	851
**Ca**	486 – 2,210	194 – 466	507 – 2,001	352 – 486	128	1,599	872
**Ti**	47.0 – 209	14.0 – 46.7	54.5 – 378	29.1 – 61.5	7.00	140	88.7
**V**	4.15 – 11.1	5.53 –12.9	3.30 – 11.4	17.8 – 27.0	4.35	8.09	5.88
**Cr**	3.37 – 8.62	4.18 – 9.77	2.90 – 9.48	4.91 – 7.40	2.11	5.92	4.26
**Mn**	15.9 – 52.3	13.0 – 19.5	17.2 – 72.7	16.8 – 24.9	3.48	44.7	28.6
**Fe**	634 – 2,233	200 – 570	706 – 3,789	414 – 614	96.8	1,648	1,023
**Ni**	2.52 – 6.84	3.37 – 5.88	2.07 – 5.25	7.74 – 11.3	1.83	4.01	3.06
**Cu**	22.3 – 46.5	35.5 – 105	14.5 – 59.8	16.5 – 94.2	20.0	19.0	23.1
**Zn**	41.4 – 187	53.9 – 97.9	35.1 – 153	90.7 – 156	21.0	99.1	104
**As**	1.00 – 2.30	1.91 – 2.73	1.13 – 1.82	7.41 – 9.23	0.98	2.73	1.72
**Sr**	3.66 – 14.0	4.94 – 7.61	4.56 – 13.4	2.10 – 3.29	1.54	12.2	8.12
**Mo**	0.84 – 3.44	1.31 – 7.88	0.49 – 2.78	1.33 – 6.01	0.74	1.61	1.72
**Cd**	0.14 – 0.59	0.43 – 0.80	0.09 – 0.38	1.31 – 1.55	0.18	0.54	0.38
**Sb**	1.05 – 4.36	1.03 – 2.81	0.63 – 2.70	3.18 – 18.3	0.70	1.73	1.24
**Ba**	7.74 – 17.6	13.2 – 18.4	6.76 – 35.3	10.4 – 53.4	4.50	21.8	14.8
**La**	0.40 – 0.82	0.03 – 0.23	0.33 – 1.62	0.42 – 0.71	0.10	1.16	0.82
**Ce**	0.74 – 1.70	0.16 – 0.41	0.70 – 3.30	0.75 – 1.21	0.15	2.30	1.53
**Nd**	0.30 – 0.75	0.06 – 0.17	0.29 – 1.37	0.30 – 0.47	0.06	0.93	0.65
**Pb**	5.63 –22.9	11.3 – 30.5	5.45 – 18.2	23.3 – 30.3	4.91	17.3	12.4

Table S2 Relative contribution of each endmember to Pb in PM_{10} collected during the local events.

		Oil combustion/refineries			Coal combustion			Sediments		
	**Site**	**Mean (%)**	**2.5%**	**97.5%**	**Mean (%)**	**2.5%**	**97.5%**	**Mean (%)**	**2.5%**	**97.5%**
**Moderate-wind-speed**	**#1**	76.9	58.8	93.1	10.0	0.4	25.8	13.1	0.5	36.0
	**#2**	73.6	53.8	91.9	11.0	0.6	27.4	15.4	0.6	40.4
	**#3**	78.5	61.0	94.2	9.3	0.4	24.1	12.2	0.4	33.8
	**#4**	70.6	50.5	88.9	12.0	0.5	28.9	17.4	0.8	44.5
	**#5**	75.4	56.9	92.7	10.5	0.5	26.6	14.1	0.6	38.5
	**#6**	73.5	54.6	91.2	11.4	0.5	28.5	15.1	0.7	40.0
**Low-wind-speed**	**#1**	77.8	59.0	94.0	9.4	0.5	24.2	12.8	0.5	35.9
	**#2**	78.6	59.9	94.5	8.6	0.4	23.4	12.7	0.6	35.2
	**#3**	80.3	61.8	95.3	8.0	0.3	21.9	11.7	0.3	33.3
	**#4**	75.3	57.3	92.6	10.6	0.5	26.7	14.0	0.6	37.4
	**#5**	88.4	73.2	98.0	4.7	0.2	14.3	6.9	0.3	21.7
	**#6**	71.3	51.3	90.0	12.7	0.5	30.1	16.0	0.6	43.0
**High-wind-speed**	**#1**	76.9	58.3	93.1	9.7	0.4	25.0	13.4	0.4	36.4
	**#2**	68.2	47.6	87.2	13.8	0.7	31.5	18.0	0.8	46.8
	**#3**	74.5	55.0	91.8	11.2	0.5	27.4	14.3	0.6	39.7
	**#4**	62.0	40.5	81.8	16.0	1.0	36.0	22.0	1.0	53.6
	**#5**	48.2	21.5	68.7	20.7	1.2	43.2	31.1	2.1	71.2
	**#6**	72.2	52.1	90.2	12.0	0.6	29.1	15.8	0.7	41.8

Table S3 Pb concentration and Pb isotope ratios of sediments collected at the Choshui River catchment in central Taiwan.

Sample name	Site	$^{206}Pb/^{204}Pb$	2 se	$^{206}Pb/^{207}Pb$	2se	$^{208}Pb/^{207}Pb$	2 se	Pb conc. (mg/kg)
**Downstream**								
**S1**	A1	18.4839	0.0015	1.1801	0.00014	2.4754	0.00032	27.4
**S2**	A1	18.4350	0.0016	1.1777	0.00016	2.4754	0.00037	24.8
**S3**	A2	18.4040	0.0020	1.1757	0.00019	2.4825	0.00043	26.3
**S4**	A2	18.5364	0.0018	1.1831	0.00016	2.4862	0.00035	21.2
**S5**	A2	18.4168	0.0016	1.1767	0.00015	2.4730	0.00033	23.5
**S6**	A2	18.3768	0.0016	1.1743	0.00015	2.4693	0.00037	27.4
**S7**	A3	18.3818	0.0017	1.1746	0.00016	2.4769	0.00036	20.9
**S8**	A4	18.4968	0.0016	1.1807	0.00014	2.4840	0.00031	20.1
**S9**	A4	18.4657	0.0014	1.1790	0.00013	2.4799	0.00030	21.1
**S10**	A5	18.4687	0.0016	1.1798	0.00014	2.4862	0.00032	25.7
**Upstream**								
**S11**	B1	18.5836	0.0014	1.1865	0.00012	2.4853	0.00027	26.0
**S12**	B2	18.6169	0.0013	1.1880	0.00012	2.4843	0.00025	23.2
**S13**	B3	18.3901	0.0013	1.1755	0.00012	2.4676	0.00027	26.7

Table S4 Method detection limits for elements determined by field blank measurements (N = 7) in this study.

**Elements**	**MDLs (ng/m^3)**	**Elements**	**MDLs (ng/m^3)**
**Na**	5.66	**Zn**	1.26
**Mg**	0.68	**As**	0.006
**Al**	6.18	**Sr**	0.02
**K**	2.67	**Mo**	0.007
**Ca**	19.0	**Cd**	0.008
**Ti**	0.78	**Sb**	0.06
**V**	0.03	**Ba**	0.23
**Cr**	0.90	**La**	0.005
**Mn**	0.25	**Ce**	0.014
**Fe**	2.86	**Nd**	0.006
**Ni**	0.24	**Pb**	0.067
**Cu**	0.76		

Table S5 Analytical results for the urban particulate matter certified reference material, NIST SRM 1648a (in μg/g, N = 3).

Element	Certified value	Measured value	Recovery (%)
**Na**	4,240 ± 60	4,596 ± 143	108.4
**Mg**	8,130 ± 120	7,740 ± 582	95.2
**Al**	34,300 ± 1300	34,066 ± 2459	99.3
**K**	10,560 ± 490	10,415 ± 643	98.6
**Ca**	58,400 ± 1900	57,704 ± 3449	98.8
**Ti**	4,021 ± 86	3,889 ± 225	96.7
**V**	127 ± 11	124 ± 7	98.0
**Cr**	402 ± 13	351 ± 15	87.4
**Mn**	790 ± 44	793 ± 21	100.4
**Fe**	39,200 ± 2100	37,209 ± 769	94.9
**Co**	17.93 ± 0.68	16.66 ± 1.0	92.9
**Ni**	81.1 ± 6.8	73.1 ± 3.7	90.1
**Cu**	610 ± 70	580 ± 44	95.1
**Zn**	4,800 ± 270	4,519 ± 283	94.1
**As**	115.5 ± 3.9	110 ± 12	95.4
**Sr**	215 ± 17	210 ± 10	97.9
**Cd**	73.7 ± 2.3	70.7 ± 4.7	96.4
**Sb**	45.4 ± 1.4	43.0 ± 2.1	94.7
**La**	39 ± 3*	35 ± 1	90.2
**Ce**	54.6 ± 2.2	49.4 ± 3.2	90.4
**Pb**	6,550 ± 330	6,254 ± 388	95.5

* Reference value (reported by NIST)

Table S6 Analytical results for Gobi Kosa dust certified reference material, NIES No. 30 (in μg/g, N = 3).

Element	Certified value	Measured value	Recovery (%)
**Na**	9,390 ± 710	9,312 ± 667	99.2
**Mg**	15,100 ± 1300	14,382 ± 997	95.2
**Al**	75,800 ± 4200	74,101 ± 3403	97.8
**K**	21,300 ± 1100	20,708 ± 1253	97.2
**Ca**	42,500 ± 3500	39,482 ± 1700	92.9
**Ti**	4,260 ± 400	4,155 ± 158	97.5
**Cr**	57.4*	59.2 ± 0.3	103.2
**Mn**	768 ± 83	702 ± 17	91.4
**Fe**	38,400 ± 3500	34,061 ± 914	88.7
**Co**	13.7*	12.2 ± 0.4	88.8
**Ni**	29.1*	26.7 ± 0.6	91.9
**Cu**	34.1*	31.5 ± 2.3	92.5
**Zn**	93.1 ± 8.5	84.1 ± 2.4	90.3
**Sr**	250 ± 20	224 ± 8	89.6
**Ba**	535 ± 31	505 ± 10	94.4
**La**	40.4*	38.8 ± 1.5	96.0
**Pb**	22.4 *	22.0 ± 0.8	98.4

* Reference values (reported by NIES[1]).

Table S7 Long-term precision and accuracy of MC-ICP-MS measurements for Pb isotope ratios at AS-IES.

Reference Material **Pb isotope ratio**	**Recommended Value (2SD)**	**Measured Value (2SD)**	**N**
NIST SRM 981			
**(^{206}Pb/^{207}Pb)**	1.0934 (0.0005)[a]	1.0932 (0.0002)	69
**(^{208}Pb/^{207}Pb)**	2.3691 (0.0011)[a]	2.3693 (0.0003)	69
NIST SRM 1648a			
**(^{206}Pb/^{207}Pb)**	1.2394[b]	1.2394 (0.0007)	12
**(^{208}Pb/^{207}Pb)**	2.4735[b]	2.4720 (0.009)	12

[a]Pb isotope ratios compiled from measurements on TIMS and MC-ICP-MS[2].

[b]Pb isotope ratios reported by Kumar *et al.*[3]

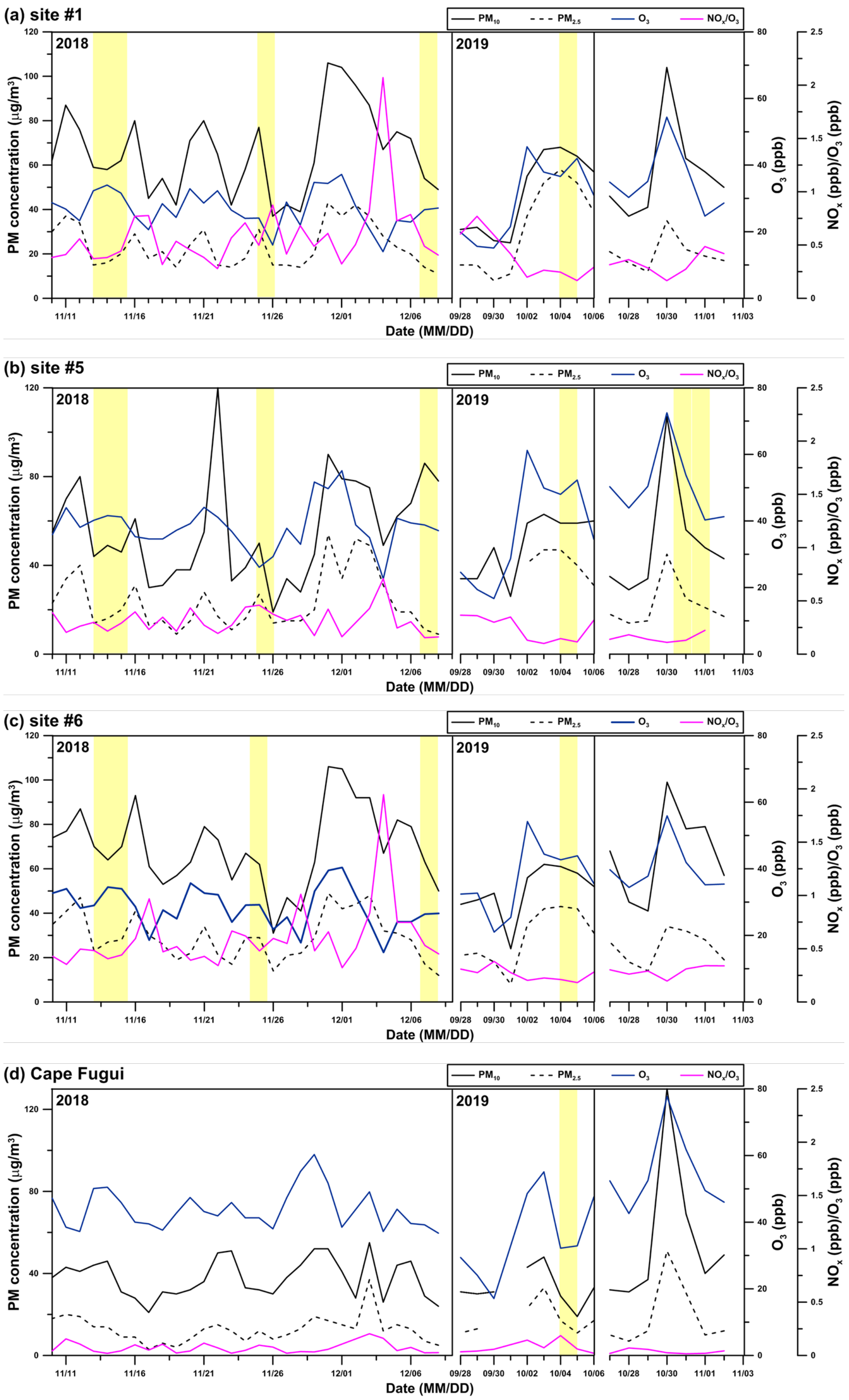

Figure S1. Daily record (Local time) of PM_{10}, $PM_{2.5}$, O_3, and the ratio of NO_x/O_3 at (a) site #1, (b) site #5, (c) site #6, and (d) Cape Fugui (available from the TEPA environment resource database). Yellow areas represent PM_{10} sampling periods conducted in this study.

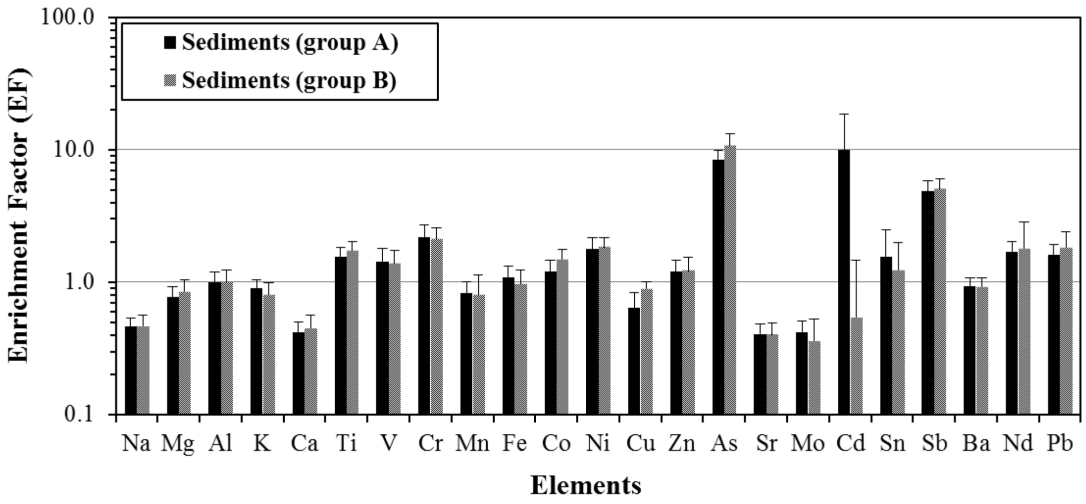

Figure S2. Enrichment factors of elements in sediments collected in the downstream (group A, N = 10) and upstream (group B, N = 3) of the Choshui River catchment in central Taiwan.

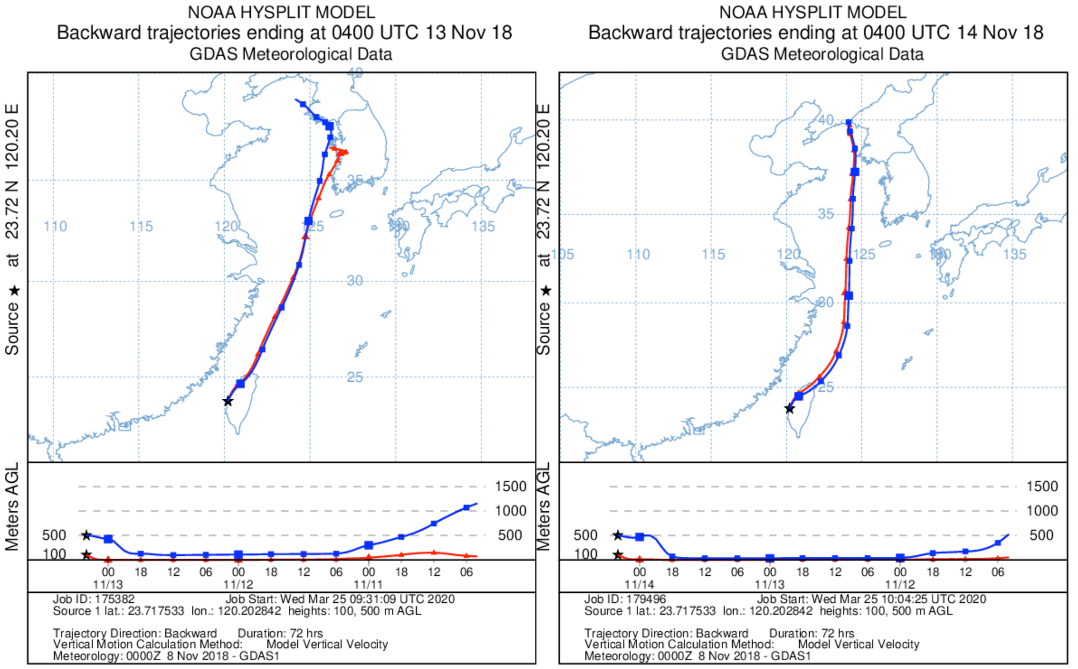

(a) Nov. 13-15, 2018

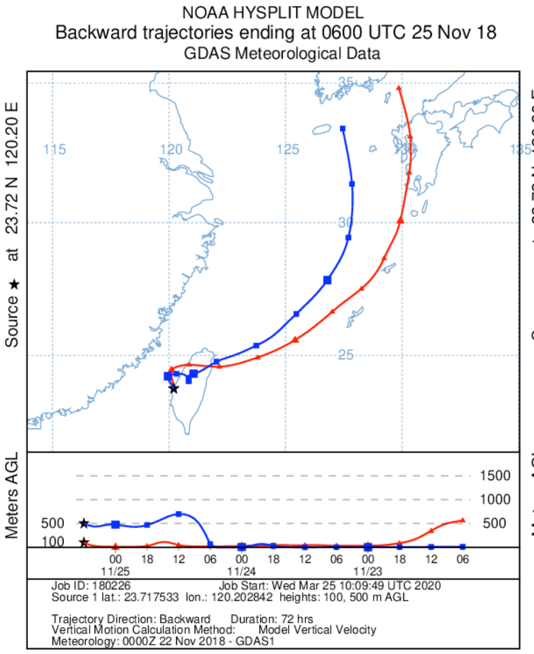

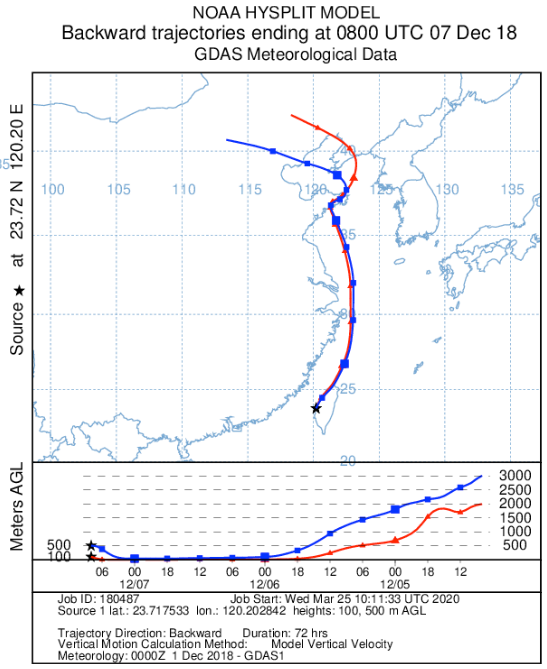

(b) Nov. 25, 2018 **(c) Dec. 7, 2018**

Figure S3. 72-hour back trajectories of air masses for (a) the moderate-wind-speed local event, (b) the low-wind-speed local event, and (c) the high-wind-speed local event in the study region in central Taiwan. Trajectories were calculated using the hybrid single-particle Lagrangian trajectory (HYSPLIT) model (NOAA Air Resources Laboratory; https://www.ready.noaa.gov/HYSPLIT.php).

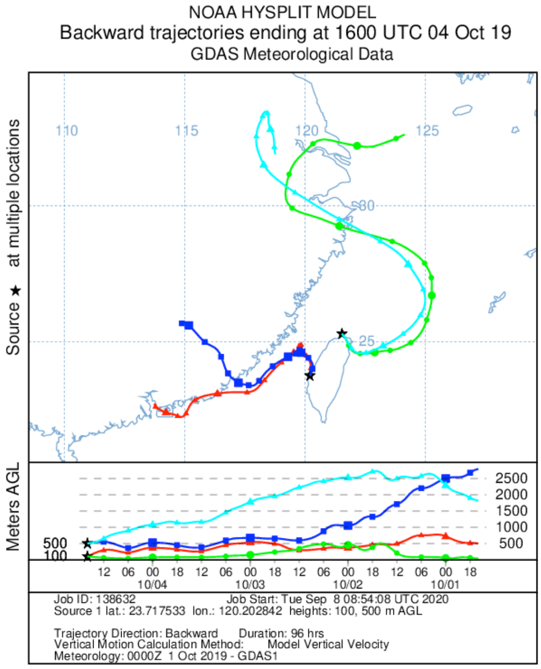

(a) Oct. 4-5, 2019

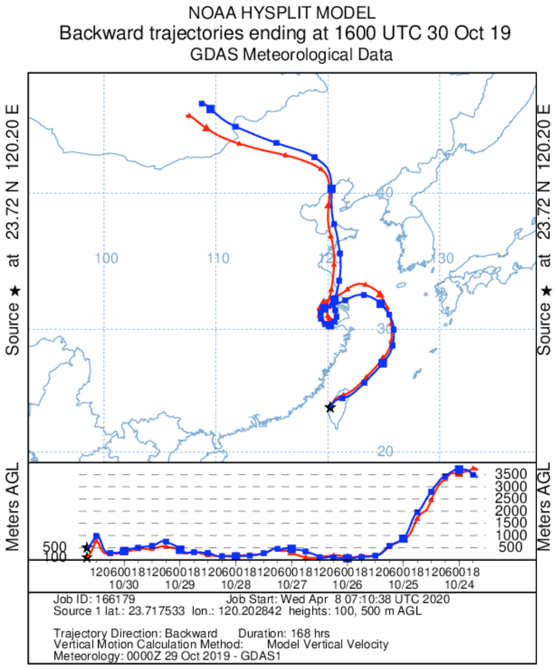

(b) Oct. 30-31, 2019

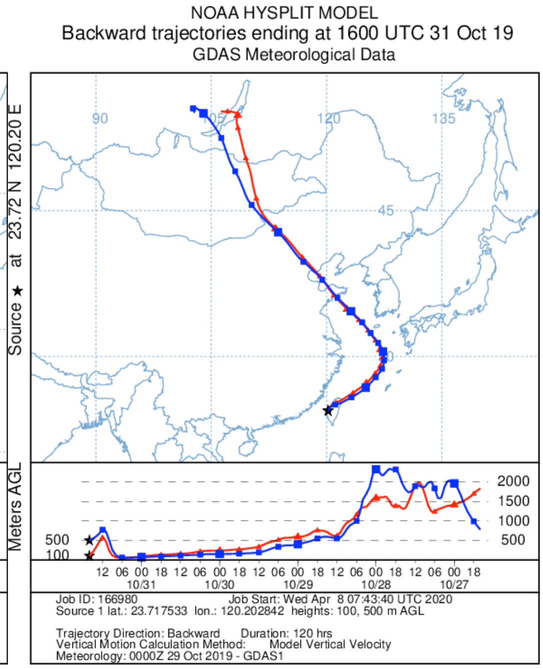

(c) Oct. 31–Nov. 1, 2019

Figure S4. (a) 96-hour back trajectories of air masses at central Taiwan and Cape Fugui during the long-range transport event. (b) 168-hour back trajectories of air masses at central Taiwan during dust storm event and (c) 144-hour back trajectories of air masses at central Taiwan during transition. Trajectories were calculated using the hybrid single-particle Lagrangian trajectory (HYSPLIT) model (NOAA Air Resources Laboratory; https://www.ready.noaa.gov/HYSPLIT.php).

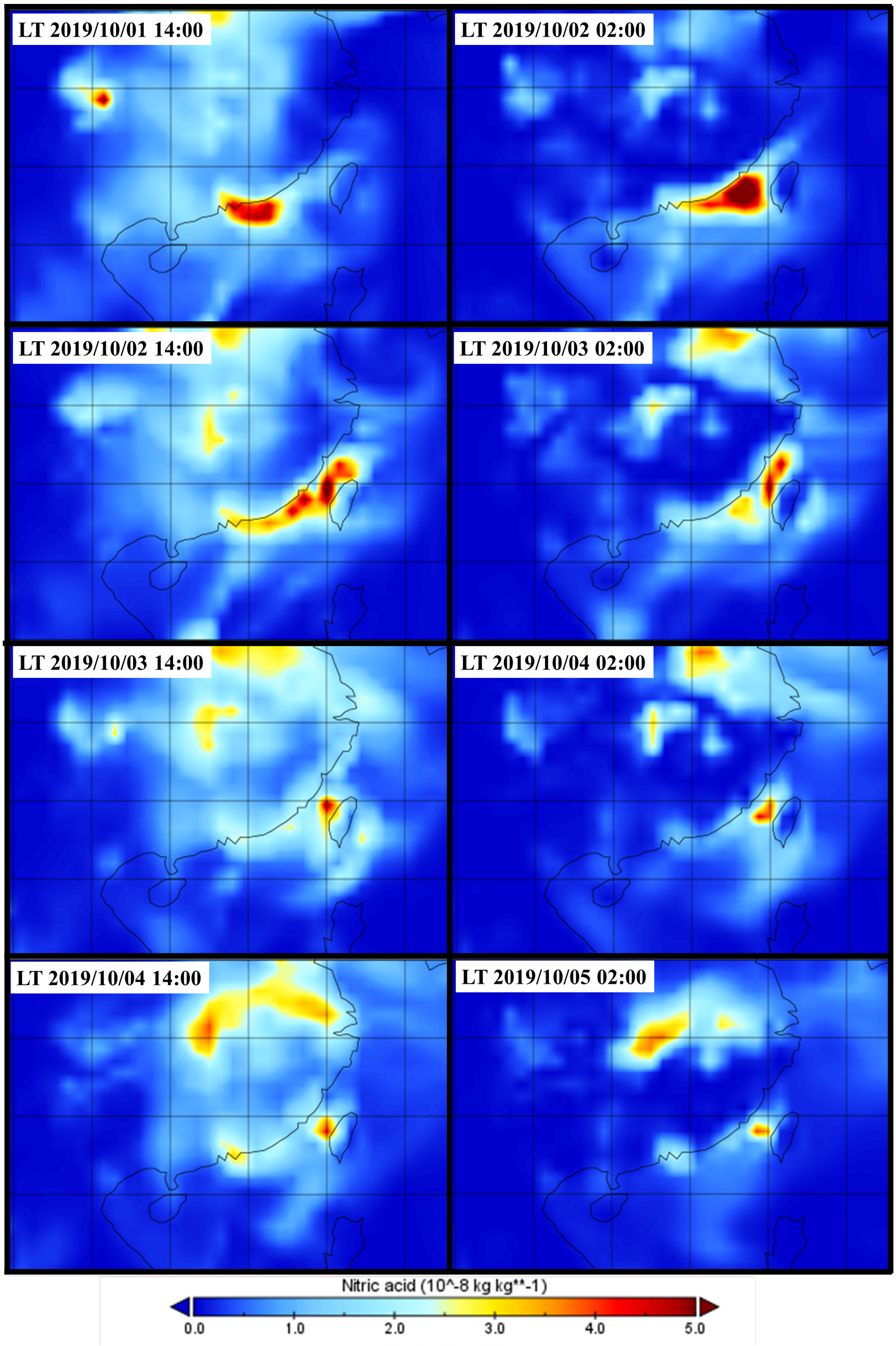

Figure S5. Nitric acid mixing ratio at the surface layer (1000 hPa) at local time (LT) during the long-range transport event in central Taiwan in 2019. Data was obtained from CAMS global reanalysis dataset (EAC4) in ECMWF (https://www.ecmwf.int/en/forecasts/dataset/cams-global-reanalysis).

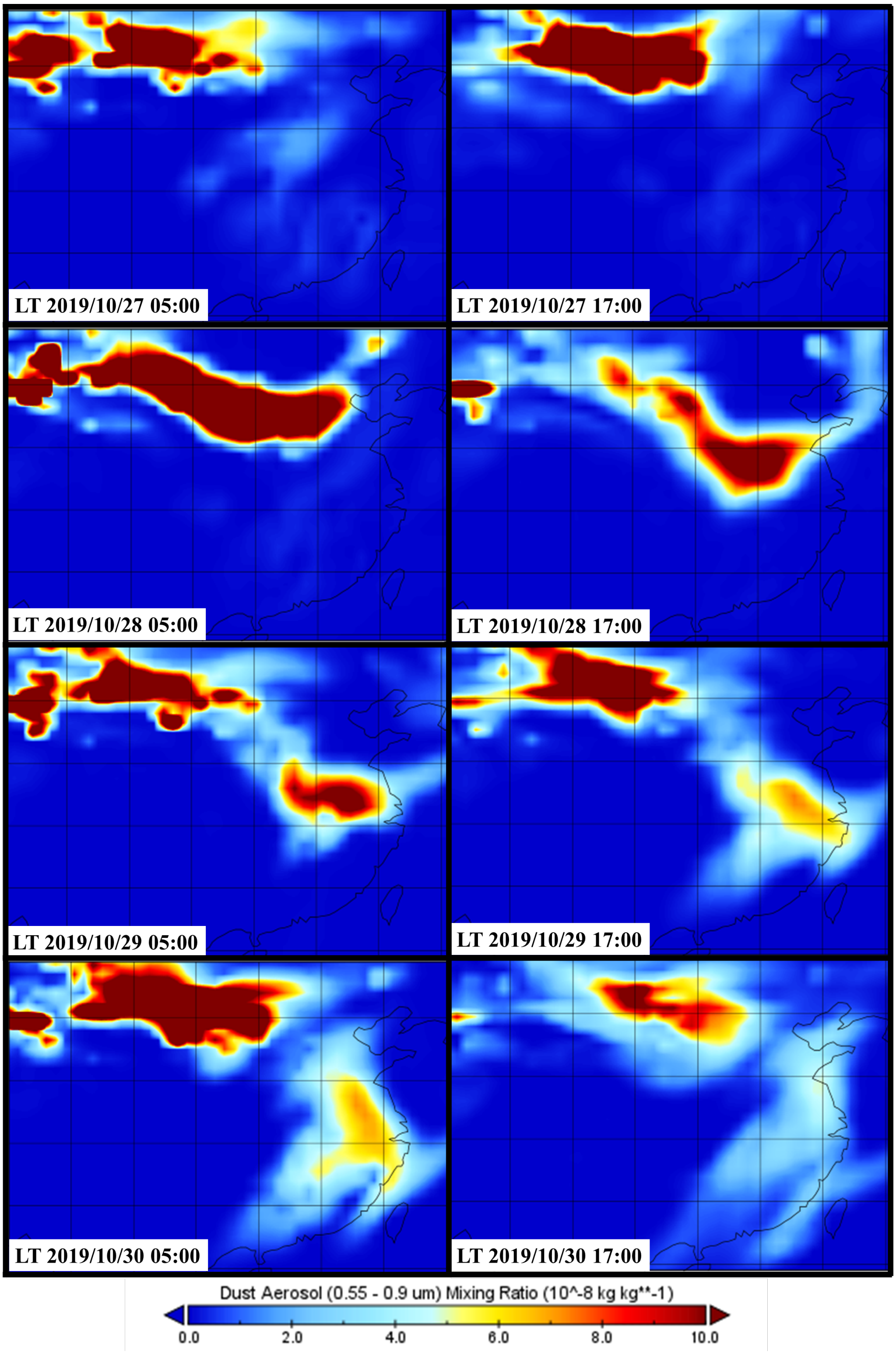

Figure S6. Dust aerosol (0.55 – 0.9 μm) mixing ratio at the surface layer (950 hPa) at local time (LT) during the dust storm event in central Taiwan in 2019. Data was obtained from CAMS global reanalysis dataset (EAC4) in ECMWF (https://www.ecmwf.int/en/forecasts/dataset/cams-global-reanalysis).

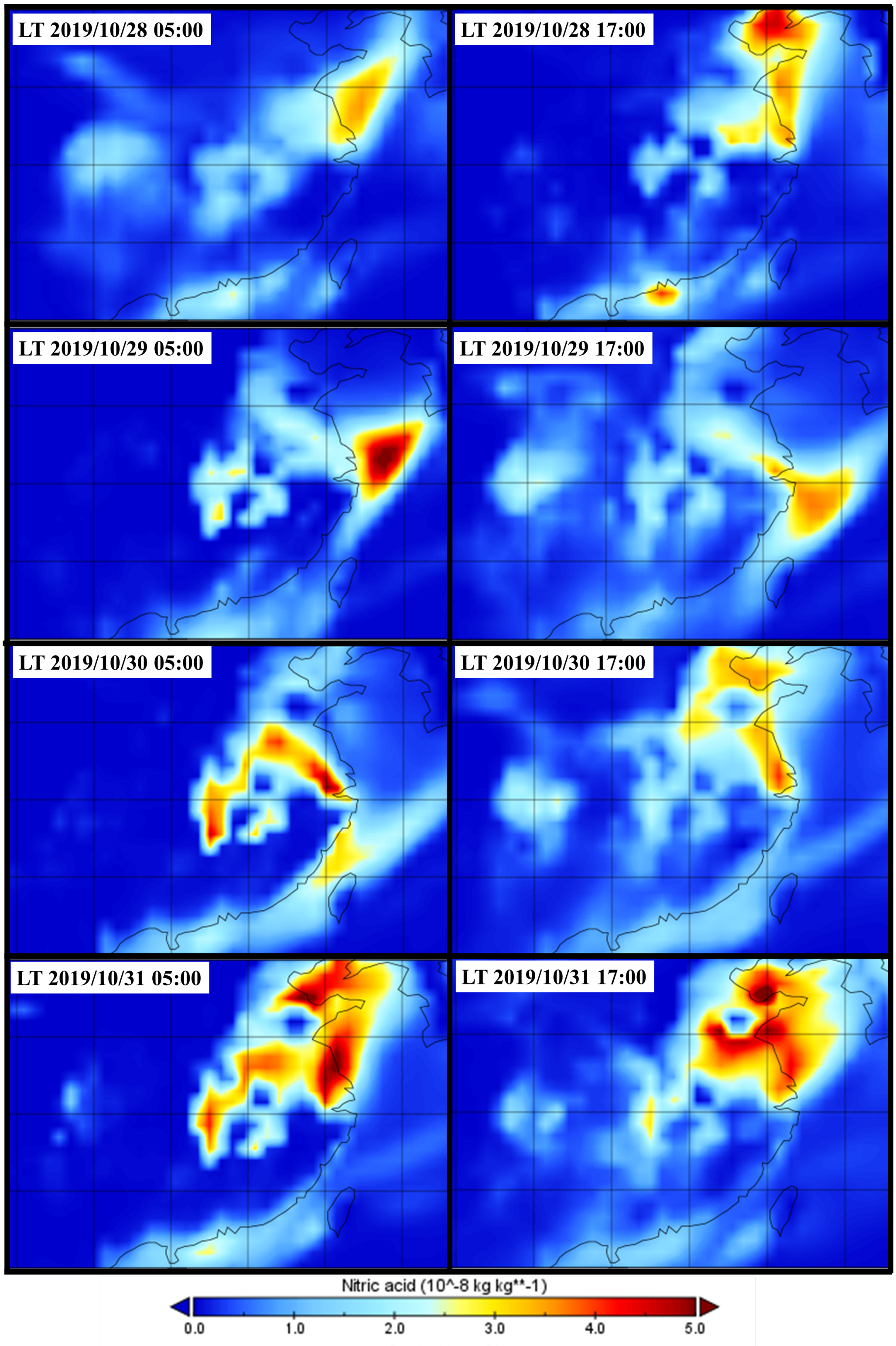

Figure S7. Nitric acid mixing ratio at the surface layer (1000 hPa) at local time (LT) during the dust storm event in central Taiwan in 2019. Data was obtained from CAMS global reanalysis dataset (EAC4) in ECMWF (https://www.ecmwf.int/en/forecasts/dataset/cams-global-reanalysis).

References:

1 Nishikawa, M. *et al.* Preparation and chemical characterisation of an Asian mineral dust certified reference material. *Analytical Methods* **5**, 4088-4095 (2013).

2 Weiss, D. J. *et al.* Accurate and precise Pb isotope ratio measurements in environmental samples by MC-ICP-MS. *International Journal of Mass Spectrometry* **232**, 205-215 (2004).

3 Kumar, S. *et al.* Understanding the Influence of Open-waste Burning on Urban Aerosols using Metal Tracers and Lead Isotopic Composition. *Aerosol and Air Quality Research* **18**, 2433-2446 (2018).
